# Identification of a Functional Genetic Variant at 16q12.1 for Breast Cancer Risk: Results from the Asia Breast Cancer Consortium

**DOI:** 10.1371/journal.pgen.1001002

**Published:** 2010-06-24

**Authors:** Jirong Long, Qiuyin Cai, Xiao-Ou Shu, Shimian Qu, Chun Li, Ying Zheng, Kai Gu, Wenjing Wang, Yong-Bing Xiang, Jiarong Cheng, Kexin Chen, Lina Zhang, Hong Zheng, Chen-Yang Shen, Chiun-Sheng Huang, Ming-Feng Hou, Hongbing Shen, Zhibin Hu, Furu Wang, Sandra L. Deming, Mark C. Kelley, Martha J. Shrubsole, Ui Soon Khoo, Kelvin Y. K. Chan, Sum Yin Chan, Christopher A. Haiman, Brian E. Henderson, Loic Le Marchand, Motoki Iwasaki, Yoshio Kasuga, Shoichiro Tsugane, Keitaro Matsuo, Kazuo Tajima, Hiroji Iwata, Bo Huang, Jiajun Shi, Guoliang Li, Wanqing Wen, Yu-Tang Gao, Wei Lu, Wei Zheng

**Affiliations:** 1 Division of Epidemiology, Department of Medicine, Vanderbilt Epidemiology Center, Vanderbilt-Ingram Cancer Center, Vanderbilt University School of Medicine, Nashville, Tennessee, United States of America; 2 Department of Biostatistics, Vanderbilt University School of Medicine, Nashville, Tennessee, United States of America; 3 Shanghai Center for Disease Control and Prevention, Shanghai, China; 4 Department of Epidemiology, Shanghai Cancer Institute, Shanghai, China; 5 Department of Epidemiology and Biostatistics, Tianjin Medical University Cancer Institute and Hospital, Tianjin, China; 6 Institute of Biomedical Sciences, Academia Sinica, Taipei, Taiwan; 7 Department of Epidemiology and Biostatistics, Nanjing Medical University, Nanjing, China; 8 Division of Surgical Oncology, Vanderbilt-Ingram Cancer Center, Vanderbilt University School of Medicine, Nashville, Tennessee, United States of America; 9 Department of Pathology, Li Ka Shing Faculty of Medicine, University of Hong Kong, Hong Kong, China; 10 Department of Preventive Medicine, Keck School of Medicine, University of Southern California/Norris Comprehensive Cancer Center, Los Angeles, California, United States of America; 11 Epidemiology Program, Cancer Research Center, University of Hawaii, Honolulu, Hawaii, United States of America; 12 Epidemiology and Prevention Division, Research Center for Cancer Prevention and Screening, National Cancer Center, Tokyo, Japan; 13 Department of Surgery, Nagano Matsushiro General Hospital, Nagano, Japan; 14 Division of Epidemiology and Prevention, Aichi Cancer Center Research Institute, Nagoya, Japan; 15 Department of Breast Oncology, Aichi Cancer Center Central Hospital, Nagoya, Japan; Georgia Institute of Technology, United States of America

## Abstract

Genetic factors play an important role in the etiology of breast cancer. We carried out a multi-stage genome-wide association (GWA) study in over 28,000 cases and controls recruited from 12 studies conducted in Asian and European American women to identify genetic susceptibility loci for breast cancer. After analyzing 684,457 SNPs in 2,073 cases and 2,084 controls in Chinese women, we evaluated 53 SNPs for fast-track replication in an independent set of 4,425 cases and 1,915 controls of Chinese origin. Four replicated SNPs were further investigated in an independent set of 6,173 cases and 6,340 controls from seven other studies conducted in Asian women. SNP rs4784227 was consistently associated with breast cancer risk across all studies with adjusted odds ratios (95% confidence intervals) of 1.25 (1.20−1.31) per allele (*P* = 3.2×10^−25^) in the pooled analysis of samples from all Asian samples. This SNP was also associated with breast cancer risk among European Americans (per allele OR  = 1.19, 95% CI  = 1.09−1.31, *P* = 1.3×10^−4^, 2,797 cases and 2,662 controls). SNP rs4784227 is located at 16q12.1, a region identified previously for breast cancer risk among Europeans. The association of this SNP with breast cancer risk remained highly statistically significant in Asians after adjusting for previously-reported SNPs in this region. *In vitro* experiments using both luciferase reporter and electrophoretic mobility shift assays demonstrated functional significance of this SNP. These results provide strong evidence implicating rs4784227 as a functional causal variant for breast cancer in the locus 16q12.1 and demonstrate the utility of conducting genetic association studies in populations with different genetic architectures.

## Author Summary

Breast cancer is one of the most common malignancies among women worldwide. Genetic factors play an important role in the etiology of breast cancer. To identify genetic susceptibility loci for breast cancer, we performed a genome-wide association study in 15,468 breast cancer cases and 13,001 controls. A single nucleotide polymorphism (SNP) rs4784227 located on chromosome 16q12.1, a previously-reported region for breast cancer risk, was found to be associated with breast cancer risk. The association of this SNP with breast cancer risk remained highly significant in Asians after adjusting all previously-reported SNPs in this region. *In vitro* biochemical experiments using both luciferase reporter and electrophoretic mobility shift assays confirmed the functional importance of this SNP. Our results demonstrate the importance of conducting genetic association studies in populations with different genetic backgrounds to identify functional variants.

## Introduction

Breast cancer is the most common malignancy among women in the United States and many other parts of the world. Genetic factors play an important role in the etiology of breast cancer. Only a very small fraction of cases in the general population, however, can be explained by high-penetrance breast cancer susceptibility genes, such as *BRCA1* and *BRCA2*. Recent genome-wide association (GWA) studies [1]–[8], including our own study among Chinese women in Shanghai [6], have identified multiple common genetic susceptibility loci for breast cancer. Each of the common genetic factors identified thus far confer only a small to moderate risk for breast cancer. With the exception of our study, all other reported GWA studies have been conducted among women of European ancestry. GWA studies conducted in other populations could identify not only additional novel genetic variants for breast cancer but also help to fine map causal variants for regions reported from previous GWA studies.

In early 2009, we reported a novel genetic susceptibility locus at 6q25.1 for breast cancer risk in a fast-track replication of promising SNPs selected from a GWA scan of 1,505 cases and 1,522 controls recruited in the Shanghai Breast Cancer Study (SBCS) [6]. We have since increased the sample size for the initial GWA scan to 2,073 cases and 2,084 controls to increase the statistical power to identify novel genetic risk variants for breast cancer. We have recently completed the second fast-track replication using data and biological samples collected from 13,395 cases and 10,917 controls recruited in 12 studies of Asian and European ancestry. SNP rs4784227, located at 16q12.1, a region identified from a previous GWA study conducted in Europeans [1], [3], was found to be a risk variant for breast cancer in Asian women independent of SNPs reported from the previous study [1], [3]. *In vitro* experimental results provide strong support for the functional significance of this SNP and suggest that this SNP may explain the association observed for breast cancer in this locus. Herein, we report findings from this large genetic study of breast cancer.

## Methods

### Ethics statement

Approval was granted from relevant review boards in all study sites; all included subjects gave informed consent.

### Study population

Included in this consortium project were 15,468 cases and 13,001 controls from 12 studies (Table 1). Detailed descriptions of these participating studies and demographic characteristics of study participants are provided in the supplement Text S1 and Table S1. Briefly, the consortium included 19,796 Chinese women from seven studies conducted in Shanghai [6], [9] (three studies, n = 10,497), Tianjin [10] (n = 3,115), Nanjing [11], [12] (n = 2,885), Taiwan [13] (n = 2,131), and Hong Kong [14] (n = 1,168); 3,214 Japanese women from three studies conducted in Hawaii [15] (n = 1,120), Nagoya [16] (n = 1,288), and Nagano (n = 806) [17]; and 5,459 European Americans from the Nashville Breast Health Study (NBHS, n = 3,172) and the Nurses' Health Study (NHS, n = 2,287, included as part of the Cancer Genetic Markers of Susceptibility Project - CGEMS). All cases and controls recruited in the Shanghai studies were included in Stages I and II, and subjects from the remaining Asian studies were included in Stage III. Data from CGEMS were used for help to select SNPs for Stage II. Cases and controls recruited in NBHS and the NHS (CGEMS) were included in the final stage to evaluate the generalizability of the findings.

**Table 1:** Characteristics of study participants and number of SNPs analyzed in each stage.

	Cases		Controls	
Study population	N	Age^c^	N	Age
Stage I (684,457 SNPs)^a^				
Shanghai – SBCS (Chinese)	2,073	49.3±8.3	2,084	49.4±8.5
Stage II (53 SNPs)				
Stage II total	4,425	53.9±10.2	1,915	52.8±9.2
Shanghai -SBCS (Chinese)	972	50.4±8.3	1,001	50.9±9.3
Shanghai - SBCSS (Chinese)	3,453	54.9±10.5		
Shanghai - SECS (Chinese)			914	54.9±8.5
Stage III (4 SNPs)^b^				
Stage III total	6,173	52.6±11.5	6,340	51.2±10.9
Tianjin (Chinese)	1,532	51.7±11.4	1,583	51.9±10.5
Nanjing (Chinese)	1,446	51.5±11.4	1,439	51.3±11.2
Taiwan (Chinese)	1,066	51.5±10.7	1,065	47.5±10.1
Hong Kong (Chinese)	517	45.8±9.5	651	45.6±10.3
Nagoya - Japan (Japanese)	644	51.4±11.0	644	51.1±10.9
Nagano - Japan (Japanese)	403	53.7±10.5	403	53.9±10.2
Hawaii - MEC (Japanese)	565	65.2±8.4	555	60.4±8.4
European Americans b				
NBHS	1,652	54.9±10.2	1,520	52.2±11.0
CGEMS	1,145		1,142	
Total	15,468	52.5±10.7	13,001	51.2±10.2
Chinese	11,059	51.8±10.3	8,737	50.4±10.1
Japanese	1,612	56.8±11.8	1,602	55.0±10.7
European Americans	2,797		2,662	

**a** Selected from SNPs included in the Affymetrix 6.0 SNP array with MAF≥1%, call rate≥95%, and QC consistency.

**b** With the exception of studies in Nanjing (2 SNPs), Tianjin (3 SNPs) and Nagoya Japan (1 SNP), NBHS (3 SNPs), CGEMS (2 SNPs genotyped and 2 SNPs imputed), four SNPs were analyzed in all other studies.

**c** Mean ± SD.

### Genotyping and quality control procedures

Genotyping for Stage I has been described previously [6]. Briefly, the initial 300 subjects were genotyped using the Affymetrix GeneChip Mapping 500 K Array Set and the remaining 3,918 subjects were genotyped using the Affymetrix Genome-Wide Human SNP Array 6.0. We included one negative control and three positive quality control (QC) samples from the Coriell Cell Repositories (http://ccr.coriell.org/) in each of the 96-well plates for Affymetrix SNP Array 6.0 genotyping. A total of 127 positive QC samples were successfully genotyped and the average concordance rate was 99.9% with a median value of 100%. The sex of all study samples was confirmed to be female. The identity-by-descent analysis based on identity-by-state was conducted to detect first-degree cryptic relationships using PLINK, version 1.06. All samples with a call rate <95% were excluded. The SNPs were excluded if: (i) minor allele frequency (MAF) <1%, (ii) call rate <95%, or (iii) genotyping concordance rate <95% in quality control samples. The final dataset included 2,073 cases and 2,084 controls for 684,457 markers.

Genotyping for Stage II was completed using the iPLEX Sequenom MassArray platform. Included in each 96-well plate as QC samples were two negative controls (water), two blinded duplicates, and two samples from the HapMap project. To compare the consistency between the Affymetrix and Sequenom platforms, we also included 124 samples from Stage I that were genotyped by Affymetrix SNP 6.0. The mean concordance rate was 99.7% for the blind duplicates, 98.8% for HapMap samples, and 98.6% between Sequenom and Affymetrix 6.0 genotyping.

Genotyping for Stage III and NBHS was performed using TaqMan at five different centers. The genotyping assay protocol was developed and validated at the Vanderbilt Molecular Epidemiology Laboratory, and TaqMan genotyping assay reagents were provided to investigators from the Tianjin study (Tianjin Cancer Institute and Hospital), Nanjing study (Nanjing Medical University), Multiethnic Cohort Study (MEC, University of Southern California), and Nagano Breast Cancer study (Japan National Cancer Center), who conducted the genotyping assays at their own laboratories. Samples from the four other studies (Hong Kong, Taiwan, Nagoya, and Nashville) were genotyped at the Vanderbilt Molecular Epidemiology Laboratory. During the genotyping, two negative controls were included in each 96-well plate, along with 30 unrelated European and 45 Chinese samples from the HapMap project genotyped together with each study for QC purposes. The consistency rate was 100.0% for the HapMap samples comparing genotyping data obtained from the current study with data obtained in the HapMap project. Each of the non-Vanderbilt laboratories was asked to genotype a trial plate containing DNA from 70 Chinese samples before genotyping study samples. The consistency rate across all centers for these trial samples was 100% compared with genotypes previously determined at Vanderbilt. In addition, replicate samples comparing 3–7% of all study samples were dispersed among the genotyping plates at all centers.

### Plasmid constructs and luciferase reporter assays

A 3.0 kb DNA fragment containing major allele (C) of rs4784227 was PCR amplified by using forward primer 5′-GATCAGCTAGCCATAGTGTGGTAGCTAGTTG-3′ and backward primer 5′-GATCA CTCGAGCTGCTGGGCTTAGCTACAAG-3′. This fragment was subcloned into luciferase reporter vector, pGL3 basic, pGL3 promoter, and pGL3 enhancer (Promega, WI) between *Nhe*1 and *Xho*1 restriction sites, respectively. The minor allele (T) sequence was generated by using QuickChange Site-Directed Mutagenesis Kit (Strategene, La Jolla, CA) with the following pair of oligonucleotides, 5′- GAGTATTTACATCACAATAATCAGCAAACACTACAAATTGGGAC-3′ and 5′- GTCCCAATTTGTAGTGTTTGCTGATTATTGTGATGTAAATACTC-3′. All DNA constructs were verified by sequencing analyses. Transfection was performed with the use of FuGene 6 Transfection Reagent (Roche Diagnostics, Indianapolis, IN) in triplicate for each of the constructs. Briefly, 1−2×10^5^ cells of HEK 293, MCF-7, MCF10A, and MDA-231 cells were seeded in 24-well plates and co-transfected with pGL4.73, a Renilla expressing vector which served as a reference for transfection efficiency. Thirty-six to 48 hours later the cells were lysed with Passive Lysis Buffer, and luminescence (relative light units) was measured using the Dual-Luciferase Assay System (Promega, WI). The rs4784227 regulatory activity was measured as a ratio of firefly luciferase activity to *renilla* luciferase activity, and the mean from four independent experiments are presented.

### Electrophoretic mobility shift assay

Biotin-labeled, double stranded oligonucleotide probes 5′-ATTTGTAGTGTTTGCCGATTATTGTGATGT-3′ and 5′-ACATCACAATAATCGGCAAACACTACAAAT-3′, and 5′-ATTTGTAGTGTTTGCTGATTATTGTGATGT-3′and 5′- ACATCACAATAATCAGCAAACACTACAAAT-3′ containing the major and minor allele sequence of rs4784227 were synthesized. The probes were incubated with nuclear protein extracts from MCF10A, MCF7, and MDA-MB-231 cells, in the presence or absence of competitors, i.e. unlabelled probes. Protein-DNA complexes were resolved by polyacrylamide gel electrophoresis and detected using a LightShift Chemiluminescent EMSA kit (Pierce Biotechnology, Rockford, IL).

### Statistical analyses

In Stage I, PLINK version 1.06 was used to analyze genome-wide data. Population structure was investigated by using the principal component analysis implemented in EIGENSTRAT [18] (http://genepath.med.harvard.edu/~reich/Software.htm). A set of 12,533 SNPs with MAF≥5% in Chinese and a distance ≥25 kb between two adjacent SNPs was selected to evaluate the population structure. The first two principal components were included in logistic regression models for adjustment of population structures. Odds ratios (OR) and 95% confidence intervals (CIs) were estimated by logistic regression analysis. ORs were also estimated for the variant allele based on a log-additive model. Age was adjusted for in the analyses of Stages I and II data. In Stage III, individual data were obtained from each study for a pooled analysis. ORs from multiple studies were adjusted for age and study site. Heterogeneity across studies and between ethnicities was assessed with likelihood ratio tests. Stratified analyses by ethnicity, menopausal status, and estrogen receptor (ER) status were carried out. P-values based on 2-tailed tests are presented.

Individual genotyping data from the Cancer Genetic Markers of Susceptibility (CGEMS, http://cgems.cancer.gov/data/) study were obtained through an approved data request application in order to perform meta-analyses of GWA scan data from both the Shanghai studies and the CGEMS project. Program MACH (http://www.sph.umich.edu/csg/abecasis/MACH/) was used for genotype imputation that determines the probability distribution of missing genotypes conditional on a set of known haplotypes while simultaneously estimating the fine-scale recombination map. For the Shanghai studies, imputation was based on 660,118 autosomal SNPs genotyped in Stage I that had a MAF>1% and passed the QC procedure, using the phased Asian data from HapMap Phase II (release 22) as the reference. A total of 2,272,352 SNPs showed an imputation quality score ≥0.90. The CGEMS GWA scan data were genotyped using Illumina HumanHap550 for 1,142 breast cancer cases and 1,145 controls nested within the Nurses' Health Study cohort. For CGEMS, genotypes were imputed based on 513,602 autosomal SNPs with MAF>1%, using phased CEU data from HapMap Phase II (release 22) as the reference. A total of 2,168,847 SNPs showed an imputation quality score ≥0.90. To evaluate associations between imputed SNP data and breast cancer risk, logistic regression (additive model) was used, in which SNPs were represented by the expected allele count, an approach that takes into account the degree of uncertainty in the genotype imputation.

Meta-analyses of GWA scan data for SBCS and CGEMS were conducted for 1,968,549 SNPs with a MAF ≥1% in both populations and imputation quality scores ≥0.90. Meta-analyses were performed using a weighted z-statistics method, where weights were proportional to the square root of the number of individuals in each sample and standardized such that the weights added up to one. The z-statistic summarizes the magnitude and direction of the effect relative to the reference allele. An overall z-statistic and p value were then calculated from the weighted average of the individual statistics. Calculations were implemented in the METAL package (http://www.sph.umich.edu/csg/abecasis/Metal).

#### SNP selection for validation in Stage II

In the present study, we selected 59 promising SNPs for fast-track replication in Stage II, including 4,425 cases and 1,915 controls recruited in the Shanghai studies. Only SNPs included in the Affymetrix 6.0 SNP arrays were selected for Stage II evaluation. Selection criteria for these SNPs were: (i) MAF≥5%, (ii) very clear genotyping clusters, (iii) not in strong LD (r^2^≤0.5) with any of the previously confirmed breast cancer genetic risk variants in CHB/JPT, (iv) consistent with HWE with P>0.01 in controls, (v) *P*<0.001 in the meta-analyses of CGEMS and SBCS GWA scan data for SNPs on Affymetrix 6.0 array, having the same direction of association in both studies, and *P≤*0.01 for SBCS GWA scan data.

## Results

**Figure 1:**
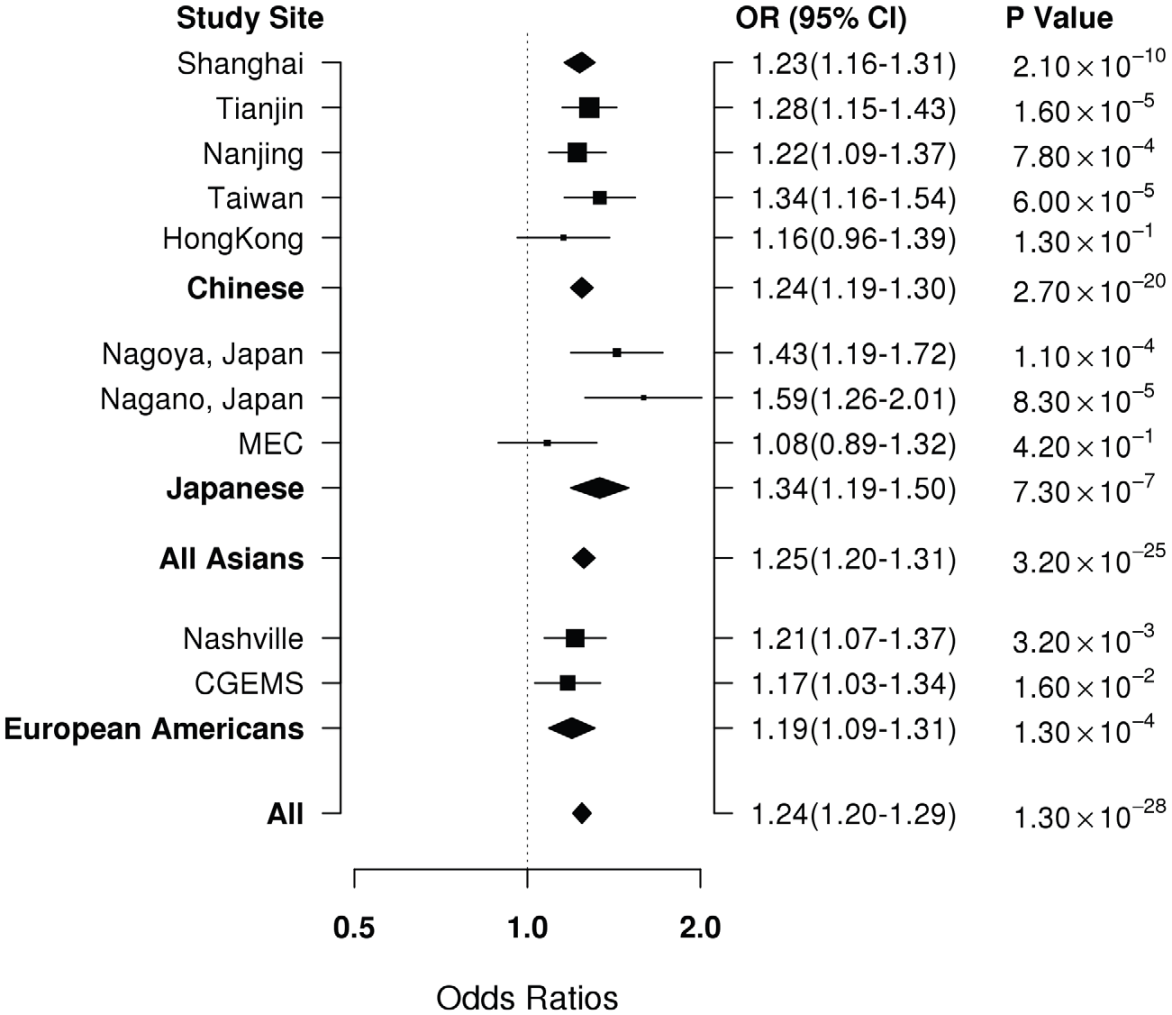
Per-allele OR for rs4784227 in association with breast cancer risk by study and ethnic groups.

Of the 53 successfully genotyped SNPs in Stage II (Table S2), highly significant associations with breast cancer risk were found for rs4784227 (16q12.1) with OR (95% CI) of 1.23 (1.16–1.31) per T allele (*P* for trend, 1.3×10^−8^) (Table 2). Three other SNPs also showed a significant or marginally significant association with breast cancer risk. These four SNPs were selected for further validation in Stage III, which included 6,173 cases and 6,340 controls of Asian ancestry from seven studies in the Asia Breast Cancer Consortium (Methods; Table S1). SNP rs4784227 was consistently associated with breast cancer risk in all studies (Figure 1), with an OR of 1.25 (95% CI: 1.20–1.31, *P* = 3.2×10^−25^) in the pooled analysis of Asian samples from all three stages. No heterogeneity in the association of this SNP with breast cancer was observed across the studies included in the consortium. The association of rs4784227 with breast cancer risk was observed in both pre-menopausal (OR: 1.24 (1.17–1.32) and *P* = 6.5×10^−12^), and post-menopausal women (OR: 1.27 (1.19–1.35) and *P* = 3.0×10^−14^) (data not shown in tables). The positive association was stronger in ER(+) breast cancer (per allele OR  = 1.29, 95% CI = 1.23–1.36, *P* = 3.0×10^−23^) than in ER(−) breast cancer (per allele OR  = 1.19, 95% CI = 1.12–1.26, *P* = 1.3×10^−8^). In case-only analyses, when compared with cases with ER(−) cancer, ORs associated with ER(+) breast cancer were found to be 1.09 (95% CI: 1.03–1.16; *P* for trend, 5.8×10^−3^). None of the other three SNPs that showed a significant association in Stage II, however, were replicated in Stage III (Table S3).

**Table 2:** Results from Stages I to III for rs4784227 and Stages I and II for other three SNPs that showed promising associations with breast cancer risk in Stage II.

						OR(95%CI)^c^			
SNP (chr)	Allele^a^	Stage	No of cases	No of controls	Frequency^b^	Heter	Homo	Per allele	P for trend
rs10479046 (5q31.1)	C/T	I	2,071	2,083	0.73/0.70	1.22(0.96–1.55)	1.42(1.12–1.80)	1.18(1.07–1.30)	7.2×10^−4^
		II	4,320	1,854	0.71/0.70	1.07(0.87–1.30)	1.15(0.94–1.40)	1.07(0.99–1.17)	0.10
		I & II	6,391	3,937	0.72/0.70	1.09(0.94–1.27)	1.22(1.06–1.42)	1.11(1.05–1.18)	7.5×10^−4^
rs3829849 (9q33.3)	T/C	I	2,067	2,081	0.11/0.09	1.31(1.12–1.54)	1.15(0.66–1.99)	1.25(1.08–1.44)	0.002
		II	4,331	1,871	0.10/0.09	1.19(1.03–1.38)	2.10(1.06–4.18)	1.23(1.08–1.41)	0.003
		I & II	6,398	3,952	0.11/0.09	1.24(1.11–1.38)	1.34(0.89–2.01)	1.22(1.11–1.34)	4.4×10^−5^
									
rs7966820 (12q24.1)	T/C	I	2,063	2,074	0.15/0.13	1.28(1.10–1.48)	1.33(0.86–2.05)	1.24(1.09–1.40)	7.8×10^−4^
		II	4,351	1,856	0.15/0.14	1.00(0.88–1.14)	2.00(1.28–3.12)	1.10(0.98–1.23)	0.10
		I & II	6,414	3,930	0.15/0.13	1.12(1.02–1.23)	1.60(1.19–2.16)	1.16(1.07–1.26)	4.0×10^−4^
rs4784227 (16q12.1)	T/C	I	2,066	2,078	0.29/0.25	1.18(1.04–1.34)	1.43(1.13–1.82)	1.19(1.08–1.31)	4.2×10^−4^
		II	4,280	1,843	0.28/0.23	1.32(1.18–1.48)	1.62(1.29–2.05)	1.30(1.19–1.42)	1.3×10^−8^
		I & II	6,346	3,921	0.28/0.24	1.24(1.14–1.35)	1.50(1.28–1.77)	1.23(1.16–1.31)	2.1×10^−10^
		I, II & III^d^	12,336	10,140	0.29/0.24	1.28(1.21–1.35)	1.52(1.37–1.69)	1.25(1.20–1.31)	3.2×10^−25^

**a** Risk allele/reference allele.

**b** Risk allele frequency in cases/controls.

**c** In Stage I, adjusted for age and the first two principal components for population structure, in Stage II adjusted for age.

**d** SNP rs4784227 was replicated in Stage III, and combined results from all three stages are presented.

**Figure 2:**
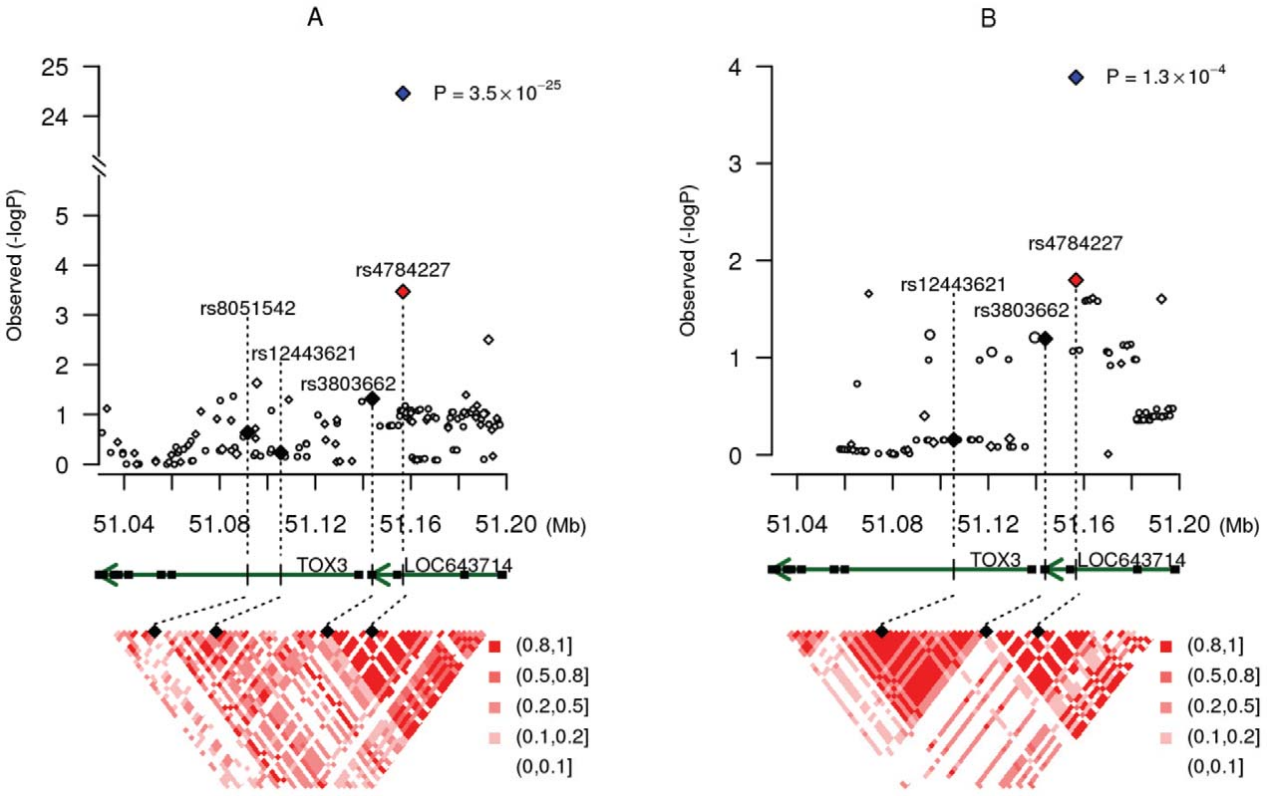
SNP association at 16q12 with breast cancer risk. In SBCS (A) and CGEMS (B). Top panel: Results (-log_10_P) are shown for the associations of breast cancer risk with directly genotyped (diamonds) and imputed (circles) SNPs located in the *TOX3* and *LOC643714* genes. P-values for rs4784227 are shown in red for Stage I data and in blue for the combined data. The three previously-reported SNPs (rs8051542, rs12443621, and rs3803662) are highlighted in black. Middle panel: Genomic view at 16q12.1. Gene locations are from the March 2006 UCSC genome browser assembly. Bottom panel: Estimates of pairwise LD (r^2^) for common SNPs (with MAF≥5%) from HapMap release 23a in the region from 10 kb downstream of rs8051542 to 10 kb upstream of rs4784227.

SNP rs4784227 is located in 16q12.1, a region where three genetic risk variants for breast cancer (rs8051542, rs12443621, and rs3803662) were reported previously in a study conducted among women of European ancestry [1]. Of these three previously reported SNPs, the closest (rs3803662) is approximately 12.8 Kb away from rs4784227. The linkage disequilibrium (LD) pattern of this region in Asians is very different from the pattern found in European descendents (Figure 2 and Table 3). In Stage I and II samples, SNP rs4784227 is in low LD with previously-reported SNPs, with r^2^ being 0.07, 0.14, and 0.37 for rs12443621, rs3803662, and rs8051542, respectively (Table 3). In European Americans included in the HapMap project, however, SNP rs4784227 is in strong LD with SNP rs3803662 (r^2^ = 0.86) but weakly correlates with the other two SNPs. SNPs rs8051542 and rs3803662 each showed a significant association with breast cancer risk (*P* = 2.0×10^−3^ and *P* = 1.7×10^−4^) in a combined analysis of data from Stages I and II (Table 4). However, after adjusting for rs4784227 the association with rs8051542 disappeared. Although the positive association with rs3803662 remained, it was of only borderline significance (*P* = 0.12). SNP rs12443621, however, showed no association with breast cancer risk, which is consistent with results reported by the initial study of Asian women [1]. The association of rs4784227 with breast cancer risk remained highly significant after adjusting for these three previously-reported SNPs, individually or in combination (Table 4). Haplotype analyses of these four SNPs showed that all haplotypes containing the T allele of rs4784227 were associated with an increased risk of breast cancer, although not all point estimates were statistically significant at *P*<0.05 due to a small sample size for several haplotypes (Table S4).

**Table 3:** Linkage disequilibrium patterns among rs4784227 and the three previously-reported SNPs in 16q12.1.

	SNPs	rs8051542	rs12443621	rs3803662	rs4784227
Chinese	rs8051542		0.1	0.08	0.37
	rs12443621	0.77		0.04	0.07
	rs3803662	0.81	0.25		0.14
	rs4784227	0.76	0.52	0.87	
					
Caucasians					
	rs8051542		0.01	0.13	0.12
	rs12443621	0.13		0.3	0.3
	rs3803662	0.52	0.92		0.86
	rs4784227	0.54	1	1	

D′: Lower left triangle.

r2: Upper right triangle.

Caucasians: European samples included in the HapMap project.

Chinese: subjects in Stages I and II.

**Table 4:** Association of rs4784227 and the three previously-reported SNPs at 16q12with breast cancer risk among Chinese women in Stages I and II.

					OR(95%CI)			
Tested SNP	Adjusted SNPs	No of cases	No of controls	Frequency^a^	Heter	Homo	Per allele	P for trend
rs4784227	None	6,346	3,921	0.28/0.24	1.24(1.14–1.35)	1.50(1.28–1.77)	1.23(1.16–1.31)	2.1×10^−10^
rs8051542	None	6,158	3,658	0.20/0.18	1.10(1.01–1.21)	1.36(1.09–1.69)	1.13(1.05–1.21)	0.002
rs12443621	None	2,954	2,997	0.57/0.57	0.99(0.86–1.14)	1.02(0.88–1.18)	1.01(0.94–1.09)	0.78
rs3803662	None	6,345	3,795	0.68/0.65	1.26(1.10–1.44)	1.34(1.17–1.53)	1.12(1.06–1.19)	1.7×10^−4^
rs4784227	rs8051542	6,082	3,614	0.28/0.24	1.27(1.14–1.40)	1.57(1.29–1.91)	1.26(1.16–1.37)	9.5×10^−8^
rs4784227	rs12443621	2,920	2,961	0.29/0.24	1.32(1.18–1.47)	1.63(1.32–2.01)	1.29(1.19–1.41)	3.1×10^−9^
rs4784227	rs3803662	6,221	3,749	0.28/0.25	1.20(1.10–1.32)	1.41(1.19–1.68)	1.20(1.11–1.28)	7.0×10^−7^
rs4784227	all 3 SNPs	2,702	2,646	0.29/0.24	1.31(1.14–1.50)	1.60(1.23–2.08)	1.28(1.15–1.44)	1.6×10^−5^
rs8051542	rs4784227	6,082	3,614	0.20/0.18	0.93(0.84–1.04)	0.97(0.76–1.25)	0.95(0.87–1.05)	0.33
rs12443621	rs4784227	2,920	2,961	0.57/0.57	0.92(0.80–1.07)	0.89(0.77–1.04)	0.95(0.88–1.02)	0.16
rs3803662	rs4784227	6,221	3,749	0.68/0.65	1.17(1.03–1.35)	1.17(1.01–1.35)	1.05(0.99–1.13)	0.12

**a** Risk allele frequency in cases/controls. Risk/reference alleles (based on forward strand) are T/C for rs8051542 and rs4784227 and A/G for rs12443621 and rs3803662.

In studies conducted in European Americans, SNP rs4784227 also showed a significant association with a per allele OR (95% CI) of 1.17 (1.03–1.34) in CGEMS and 1.21 (1.07–1.37) in NBHS (Table 5). A significant (NBHS) or marginally significant (CGEMS) association was observed for rs3803662, a previously reported SNP, but not for two other previously-reported SNPs. After adjusting for rs4784227, no association with rs3803662 was seen. On the other hand, the positive association with rs4784227 remained after adjusting for rs3803662 or the other two SNPs, although the association was no longer statistically significant at *P*<0.05.

**Table 5:** Association of rs4784227 and the three previously-reported SNPs at 16q12with breast cancer risk among European American women.

Tested SNP a	Study	SNPs Adjusted	No of cases	No of controls	Frequency^b^	OR (95% CI)	P for trend
rs4784227	CGEMS	None	1145	1142	0.29/0.25	1.17(1.03–1.34)	0.016
	NBHS	None	1357	1148	0.28/0.25	1.21(1.07–1.37)	0.003
rs3803662	CGEMS	None	1145	1142	0.3/0.27	1.13(0.99–1.28)	0.06
	NBHS	None	1615	1467	0.31/0.27	1.22(1.09–1.36)	4.0×10^−4^
rs8051542	NBHS	None	1587	1439	0.46/0.44	1.07(0.97–1.19)	0.18
rs12443621	CGEMS	None	1145	1142	0.50/0.49	1.02(0.91–1.15)	0.70
rs4784227	NBHS	rs8051542	1263	1080	0.28/0.25	1.22(1.06–1.40)	0.007
rs4784227	CGEMS	rs12443621	1145	1142	0.29/0.25	1.25(1.06–1.47)	0.007
rs4784227	Both	rs3803662	2431	2243	0.29/0.25	1.22(0.94–1.59)	0.13
rs8051542	NBHS	rs4784227	1263	1080	0.45/0.44	0.98(0.86–1.11)	0.74
rs12443621	CGEMS	rs4784227	1145	1142	0.50/0.49	0.91(0.79–1.05)	0.19
rs3803662	Both	rs4784227	2431	2243	0.31/0.27	0.97(0.75–1.25)	0.82

**a** In CGEMS, data on rs8051542 was not available, and In NBHS data on for rs12443621 was not available.

**b** Risk allele frequency in cases/controls. Risk/reference alleles (based on forward strand) are T/C for rs8051542 and rs4784227 and A/G for rs12443621 and rs3803662.

**Figure 3:**
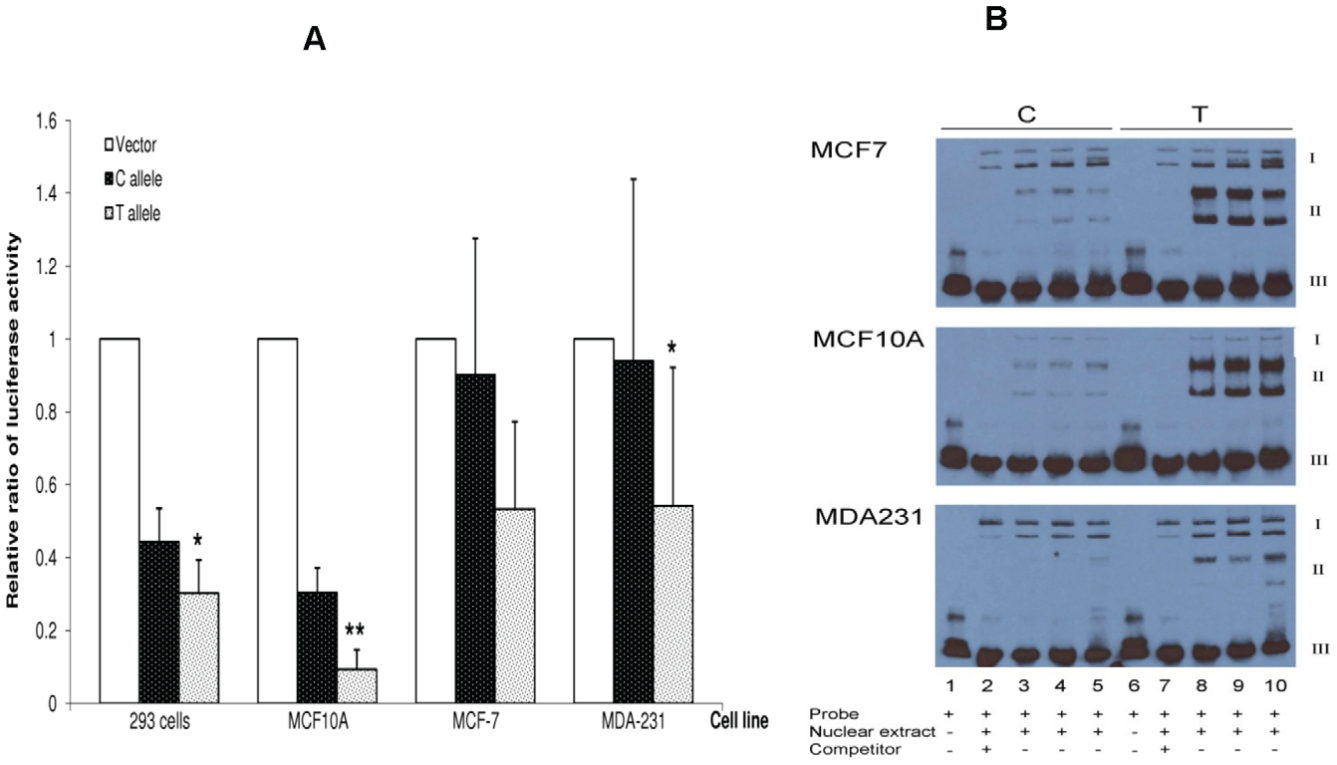
*In vitro* functional characterization of SNP rs4784227. (A) Effect of rs4784227 on luciferase reporter activity. HEK 293, MCF10A, MCF-7, and MDA-231 cells were transiently transfected with pGL3 promoter vector and the constructs carrying the reference allele (C) and risk allele (T) of rs4784227, respectively. Relative luciferase activities are shown as mean ± SD of four experiments (relative to empty vector). Statistical analysis was done using Student's t-test comparing C and T alleles (* *P*<0.05, ** *P*<0.01, n = 4). (B) Electrophoretic mobility shift assays. Nuclear protein extracts from MCF-7 (top panel), MCF10A (middle panel), and MDA-231 (bottom panel) cells were incubated with biotin-labeled probes corresponding to reference allele C (lanes 1–5) or the risk allele T (lanes 6–10) of rs4784227 in the absence or presence of competitors. Lanes 1 and 6, no nuclear extracts; lanes 2 and 7, unlabeled competitor in 200-fold molar excess; lanes 3 and 8 (5 mM MgCl2), lanes 4 and 9 (2.5 mM MgCl2), and lanes 5 and 10 (1.25 mM MgCl2), no competitor. I: nonspecific DNA-protein complex bands from MCF-7, MCF10A, and MDA-231 cells; II: specific DNA-protein complex bands; III: free biotin-labeled probes.

To evaluate whether SNP rs4784227 has any intrinsic regulatory function, we conducted an *in vitro* luciferase assay in four cell lines including metastatic breast cancer cell MDA231, non-metastatic breast cancer cell MCF-7, breast epithelial cell MCF10A, and HEK293. Luciferase reporter constructs containing a 3 kb DNA fragment with the reference allele C and the risk allele T of rs4784227, respectively, were generated and transiently transfected into these cells. By comparing to the respective empty vectors, no luciferase activity change was observed in pGL3 basic and pGL3 enhancer vectors that harbor rs4784227 fragments, which indicate that rs4784227 fragments do not have intrinsic promoter activity (data not shown). In contrast, in the pGL3 promoter vector, fragments containing rs4784227 reduced luciferase activity, and the reduction was more apparent in fragments containing risk allele T than the reference allele C (Figure 3A). With the exception of the MCF7 cells, the difference between the T and C allele was statistically significant at P≤0.05.

To investigate whether the DNA sequence containing rs4784227 may interact with nuclear proteins and if so, whether a single nucleotide change in the rs4784227 site may alter the protein-DNA interactions, we performed electrophoretic mobility shift assays. In these assays, oligonucleotide probes corresponding to the reference allele C or the risk allele T were incubated with nuclear protein extracts from MCF10A, MCF-7, and MDA-231 cells. Compared with reference allele C, risk allele T in rs4784227 resulted in an altered DNA-protein complex intensity in these cells (B and II) (Figure 3B). In contrast, risk allele T did not alter the intensity of the nonspecific DNA-protein complex band (I). These results were unaffected by the presence of large amounts of unlabeled competitors.

## Discussion

In this multi-stage GWA study of over 15,486 cases and 13,001 controls, we identified SNP rs4784227 as highly significantly associated with breast cancer in both Asians (per allele OR = 1.25, 95% CI = 1.20–1.31, *P* = 3.2×10^−25^) and European Americans (per allele OR = 1.19, 95% CI = 1.09–1.31, P = 1.3×10^−4^). SNP rs4784227 is located at 16q12.1, a region reported previously to harbor breast cancer genetic risk variants among European descendents [1], [3]. In Asians, however, this SNP is either not in LD or only in weak LD with any of the three previously-reported SNPs in these regions, and adjusting for these SNPs did not alter the association of breast cancer with this newly-identified SNP. Although in European Americans rs4784227 is in strong LD with one of the previously-reported SNPs, rs3803602, the positive association of rs4784227 with breast cancer remained after adjusting for previously-reported SNPs. *In vitro* experiments showed that risk allele *T* reduced luciferase activity and altered DNA-protein binding patterns. These results implicate rs4784227 as a functional genetic risk variant for breast cancer, and this SNP may explain, at least partially, the association of breast cancer with other SNPs identified in 16q12.1.

SNP rs4784227 is located 18.4 kb upstream of the *TOX3* gene and in the evolutionarily-conserved region of intron of the *LOC643714* gene. Several transcription factors are predicted to bind to this SNP (http://www.cbrc.jp/research/db/TFSEARCH.html). This SNP, however, has not been shown to be in the coding region for any non-coding RNA or miRNA/snoRNA/scaRNA based on UCSC Genome Browser. Our luciferase reporter assays showed that the intronic region harboring rs4784227 may have intrinsic repressor activities, suggesting that rs4784227 may affect its underlying gene *LOC643714* or its neighborhood gene expression and thus affect breast cancer risk. The rs4784227-associated repressing activity could be the result of differential binding affinity of transcription machinery to the rs4784227-containing DNA sequences. We examined this hypothesis by conducting electrophoretic mobility shift assays and confirmed that the risk *T* allele of rs4784227 significantly alter DNA-nuclear protein(s) interactions. Thus, it is possible that inhibitory nuclear protein(s) selectively bind to the risk allele *T* to repress transcription. A database search (http://www.cbrc.jp/research/db/TFSEARCH.html) for transcription factor binding sites showed that the sequence at the rs4784227 site has a high degree of similarity with several consensus elements recognized by transcription factors, of which HNF-3b and C/EBP prefer to bind DNA fragments with the risk T allele of this SNP site. However, these putative transcription factors or their associated proteins have not been confirmed to be involved in the regulation of *LOC643714* or its nearby genes.

In summary, through a GWA study we have identified and confirmed rs4784227 as a genetic risk variant for breast cancer. *In vitro* experiments showed a functional significance of this SNP that may explain the association of breast cancer with other SNPs identified at locus 16q12.1. This study demonstrates the importance of conducting genetic association studies in populations with different LD structures to identify causal genetic variants for breast cancer and other complex diseases.

## Supporting Information

Table S1 Characteristics of participating studies in the Asian Breast Cancer Consortium.
(0.06 MB DOC)

Table S2 Associations of 49 SNPs evaluated in Stage II but not in Stage III.
(0.27 MB DOC)

Table S3 Results for four SNPs selected for Stage III evaluation by study.
(0.09 MB DOC)

Table S4 Haplotype analyses of the four SNPs at 16q12 with breast cancer risk in Stages I and II.
(0.05 MB DOC)

Text S1 Study participants.
(0.11 MB DOC)
