# Supplementary material for: Identification of a Functional Genetic Variant at 16q12.1 for Breast Cancer Risk: Results from the Asia Breast Cancer Consortium
Source: PLoS Genet. 2010 Jun 24;6(6):e1001002. doi: 10.1371/journal.pgen.1001002 (PMC2891809; doi:10.1371/journal.pgen.1001002)
Supplement: Table S1 — Characteristics of participating studies in the Asian Breast Cancer Consortium. (0.06 MB DOC) [file pgen.1001002.s001.doc]

**Table S1. Characteristics of participating studies in the Asian Breast Cancer Consortium.**

| Study | Ethnicity | Study design^f^ | Study period | N^a^ | Age | Menopause (%) | ER (+)^d^ % |
| --- | --- | --- | --- | --- | --- | --- | --- |
| SBCS-I^e^ | Chinese | Population | 1996-1998 | 1,115/1,235 | 47.6/47.2 | 32.8/36.1 | 63.6 |
| SBCS-II^e^ | Chinese | Population | 2002-2005 | 1,930/1,850 | 50.9/51.7 ^b^ | 43.6/49.3 ^b^ | 63.5 |
| SBCSS^e^/SECS^e^ | Chinese | Population | 2002-2006 | 3,453/ 914 | 54.9/54.9 | 53.7/61.6 ^b^ | 64.4 |
| Tianjin | Chinese | Hospital | 2004-2008 | 1,532/1,583 | 51.7/51.9 | 51.7/55.4 ^c^ | 44.3 |
| Nanjing | Chinese | Hospital | 2004-2008 | 1,446/1,439 | 51.5/51.3 | 50.5/53.7 | 43.2 |
| Taiwan | Chinese | Hospital | 2004-2007 | 1,066/1,065 | 51.5/47.5 ^b^ | 52.3/39.9 ^b^ | 66.1 |
| Hong Kong | Chinese | Hospital | 2003-2009 | 517/ 651 | 45.8/45.6 | 50.6/41.6 ^c^ | 72.8 |
| Nagoya, Japan | Japanese | Hospital | 2003-2005 | 644/ 644 | 51.4/51.1 | 48.5/48.5 | 72.8 |
| Nagano, Japan ^e^ | Japanese | Hospital | 2001-2005 | 403/ 403 | 53.7/53.9 | 54.6/65.0 ^b^ | 74.7 |
| MEC^e^ | Japanese | Population | 1993-2008 | 565/ 555 | 65.2/60.4 ^b^ | 84.8/80.8 | 86.2 |
| NBHS | European Americans | Population | 2001-2008 | 1,652/1,520 | 54.9/52.2 | 65.5/58.9^b^ | 74.9 |
| CGEMS | European Americans | Population | 1989-2004 | 1,145/1,142 |  | 100/100 |  |

1. Cases/controls
2. Significant at α≤0.01 level (t-test for continuous variables, Chi-square test for categorical variables).
3. Significant at α≤0.05 level (t-test for continuous variables, Chi-square test for categorical variables).
4. Among cases with estrogen receptor (ER) data.
5. SBCS-I: Shanghai Breast Cancer Study-I; SBCS-II: Shanghai Breast Cancer Study-II; SBCSS: Shanghai Breast Cancer Survival Study; SECS: Shanghai Endometrial Cancer Study; Nagano Japan: Nagano Breast Cancer Study; Nagoya, Japan: Hospital-based Epidemiologic Research Program at Aichi Cancer Center; MEC: Multiethnic Cohort Study;
6. With the exception of the MEC (a cohort study), all other studies used the case-control study design with either a population-based or hospital-based approach.
