# Supplementary material for: Identification of a Functional Genetic Variant at 16q12.1 for Breast Cancer Risk: Results from the Asia Breast Cancer Consortium
Source: PLoS Genet. 2010 Jun 24;6(6):e1001002. doi: 10.1371/journal.pgen.1001002 (PMC2891809; doi:10.1371/journal.pgen.1001002)
Supplement: Table S2 — Associations of 49 SNPs evaluated in Stage II but not in Stage III. (0.27 MB DOC) [file pgen.1001002.s002.doc]

Table S2. Associations of 49 SNPs evaluated in Stage II but not in Stage III.

|  |  |  |  |  |  |  |  | OR(95%CI)^d^ | | |  |
| --- | --- | --- | --- | --- | --- | --- | --- | --- | --- | --- | --- |
| SNP ^a^ | chr | position | alleles^b^ | Population | No of cases | No of controls | frequency^c^ | Heterozygous | Homozygous | Per allele | P trend |
| rs422481 | 1 | 30301721 | A/G | I | 2073 | 2080 | 0.25/0.28 | 0.90(0.79-1.02) | 0.72(0.56-0.93) | 0.87(0.79-0.96) | 7.2 x 10^-3^ |
|  |  |  |  | II | 4089 | 1846 | 0.26/0.25 | 1.03(0.92-1.16) | 1.03(0.82-1.30) | 1.02(0.94-1.12) | 0.61 |
|  |  |  |  | Combined | 6162 | 3926 | 0.26/0.27 | 0.96(0.88-1.04) | 0.88(0.75-1.03) | 0.95(0.89-1.01) | 0.1 |
| rs12041465 | 1 | 75381637 | A/C | I | 1927 | 1935 | 0.22/0.25 | 0.78(0.68-0.89) | 0.82(0.62-1.08) | 0.84(0.75-0.93) | 9.1 x 10^-4^ |
|  |  |  |  | II | 4311 | 1837 | 0.24/0.24 | 0.97(0.86-1.09) | 1.00(0.78-1.27) | 0.98(0.90-1.08) | 0.7 |
|  |  |  |  | Combined | 6238 | 3772 | 0.23/0.25 | 0.90(0.82-0.98) | 0.92(0.77-1.10) | 0.93(0.87-0.99) | 0.02 |
| rs12118297 | 1 | 87551805 | T/G | I | 2072 | 2084 | 0.34/0.39 | 0.83(0.73-0.95) | 0.65(0.53-0.78) | 0.81(0.74-0.89) | 4.3 x 10^-6^ |
|  |  |  |  | II | 4371 | 1862 | 0.36/0.36 | 1.04(0.93-1.17) | 0.97(0.81-1.14) | 1.00(0.92-1.08) | 0.98 |
|  |  |  |  | Combined | 6443 | 3946 | 0.35/0.38 | 0.93(0.86-1.02) | 0.81(0.71-0.91) | 0.91(0.86-0.96) | 1.0 x 10^-3^ |
| rs12756720 | 1 | 164182207 | C/A | I | 2062 | 2058 | 0.17/0.14 | 1.29(1.12-1.48) | 1.05(0.69-1.59) | 1.21(1.07-1.36) | 2.2 x 10^-3^ |
|  |  |  |  | II | 4037 | 1857 | 0.12/0.13 | 0.86(0.76-0.98) | 1.46(0.40-5.31) | 0.87(0.77-0.99) | 0.03 |
|  |  |  |  | Combined | 6099 | 3915 | 0.14/0.14 | 1.00(0.92-1.10) | 0.74(0.50-1.09) | 0.98(0.90-1.07) | 0.62 |
| rs7567740 | 2 | 40005386 | C/A | I | 2071 | 2081 | 0.17/0.19 | 0.85(0.75-0.98) | 0.69(0.50-0.95) | 0.84(0.76-0.94) | 2.6 x 10^-3^ |
|  |  |  |  | II | 4345 | 1858 | 0.18/0.18 | 1.02(0.91-1.15) | 0.87(0.64-1.18) | 0.99(0.89-1.09) | 0.82 |
|  |  |  |  | Combined | 6416 | 3939 | 0.18/0.19 | 0.96(0.88-1.04) | 0.77(0.62-0.95) | 0.92(0.86-0.99) | 0.03 |
| rs10199005 | 2 | 57124711 | A/G | I | 2062 | 2067 | 0.15/0.18 | 0.87(0.76-1.00) | 0.59(0.40-0.88) | 0.84(0.74-0.94) | 2.6 x 10^-3^ |
|  |  |  |  | II | 4333 | 1850 | 0.16/0.16 | 0.99(0.87-1.12) | 0.96(0.69-1.34) | 0.99(0.89-1.09) | 0.79 |
|  |  |  |  | Combined | 6395 | 3917 | 0.16/0.17 | 0.92(0.84-1.01) | 0.81(0.64-1.04) | 0.91(0.85-0.99) | 0.02 |
| rs4516385 | 2 | 180770919 | A/G | I | 1924 | 1931 | 0.39/0.35 | 1.13(0.98-1.29) | 1.40(1.15-1.71) | 1.17(1.07-1.28) | 8.4 x 10^-4^ |
|  |  |  |  | II | 4368 | 1859 | 0.36/0.37 | 0.89(0.79-1.00) | 0.92(0.78-1.09) | 0.94(0.87-1.02) | 0.14 |
|  |  |  |  | Combined | 6292 | 3790 | 0.37/0.36 | 0.98(0.89-1.06) | 1.09(0.96-1.24) | 1.03(0.97-1.09) | 0.4 |
| rs11712576 | 3 | 27511803 | C/A | I | 1928 | 1938 | 0.11/0.09 | 1.24(1.05-1.46) | 1.31(0.72-2.41) | 1.22(1.05-1.41) | 8.1 x 10^-3^ |
|  |  |  |  | II | 4207 | 1862 | 0.11/0.10 | 1.00(0.87-1.15) | 1.15(0.66-2.02) | 1.02(0.89-1.15) | 0.82 |
|  |  |  |  | Combined | 6135 | 3800 | 0.11/0.10 | 1.10(0.99-1.22) | 1.20(0.80-1.81) | 1.10(1.00-1.21) | 0.05 |
| rs17006208 | 4 | 83879972 | C/T | I | 2071 | 2079 | 0.16/0.13 | 1.34(1.16-1.54) | 1.62(1.04-2.54) | 1.32(1.17-1.50) | 1.1 x 10^-5^ |
|  |  |  |  | II | 4348 | 1853 | 0.14/0.15 | 0.93(0.82-1.06) | 0.64(0.43-0.95) | 0.90(0.80-1.00) | 0.05 |
|  |  |  |  | Combined | 6419 | 3932 | 0.15/0.14 | 1.10(1.00-1.20) | 0.94(0.70-1.27) | 1.06(0.98-1.15) | 0.14 |
| rs2869950 | 4 | 89961787 | C/T | I | 1926 | 1938 | 0.32/0.28 | 1.16(1.02-1.33) | 1.41(1.12-1.76) | 1.18(1.07-1.30) | 9.4 x 10^-4^ |
|  |  |  |  | II | 4337 | 1844 | 0.32/0.31 | 1.02(0.91-1.14) | 1.07(0.88-1.29) | 1.03(0.95-1.12) | 0.52 |
|  |  |  |  | Combined | 6263 | 3782 | 0.32/0.3 | 1.10(1.01-1.19) | 1.21(1.05-1.40) | 1.10(1.03-1.17) | 2.7 x 10^-3^ |
| rs13112866 | 4 | 102339477 | A/G | I | 2065 | 2080 | 0.33/0.36 | 0.94(0.83-1.07) | 0.69(0.56-0.84) | 0.86(0.79-0.94) | 1.3 x 10^-3^ |
|  |  |  |  | II | 4322 | 1850 | 0.36/0.34 | 1.08(0.96-1.21) | 1.18(0.99-1.41) | 1.08(1.00-1.17) | 0.05 |
|  |  |  |  | Combined | 6387 | 3930 | 0.35/0.35 | 1.02(0.94-1.11) | 0.95(0.83-1.08) | 0.99(0.93-1.05) | 0.64 |
| rs7677505 | 4 | 118828177 | T/C | I | 2066 | 2073 | 0.40/0.35 | 1.29(1.13-1.47) | 1.47(1.22-1.78) | 1.23(1.13-1.35) | 4.9 x 10^-6^ |
|  |  |  |  | II | 4367 | 1858 | 0.38/0.38 | 1.04(0.93-1.17) | 0.99(0.84-1.16) | 1.00(0.93-1.09) | 0.91 |
|  |  |  |  | Combined | 6433 | 3931 | 0.39/0.37 | 1.13(1.03-1.23) | 1.17(1.03-1.32) | 1.09(1.03-1.16) | 2.9 x 10^-3^ |
| rs4834179 | 4 | 128244236 | A/G | I | 2060 | 2065 | 0.41/0.46 | 0.83(0.72-0.95) | 0.66(0.55-0.79) | 0.81(0.74-0.89) | 4.5 x 10^-6^ |
|  |  |  |  | II | 4353 | 1860 | 0.43/0.44 | 0.93(0.82-1.05) | 0.95(0.81-1.11) | 0.97(0.90-1.04) | 0.39 |
|  |  |  |  | Combined | 6413 | 3925 | 0.42/0.45 | 0.87(0.79-0.95) | 0.81(0.73-0.91) | 0.90(0.85-0.95) | 2.2 x 10^-4^ |
| rs13131392 | 4 | 128290321 | A/G | I | 2063 | 2062 | 0.41/0.46 | 0.78(0.68-0.90) | 0.66(0.55-0.79) | 0.81(0.74-0.88) | 3.4 x 10^-6^ |
|  |  |  |  | II | 4362 | 1859 | 0.43/0.44 | 0.97(0.86-1.09) | 0.96(0.82-1.12) | 0.98(0.90-1.05) | 0.55 |
|  |  |  |  | Combined | 6425 | 3921 | 0.43/0.45 | 0.86(0.79-0.94) | 0.82(0.73-0.92) | 0.90(0.85-0.95) | 3.4 x 10^-4^ |
| rs9307594 | 4 | 128313759 | A/G | I | 2038 | 2019 | 0.15/0.18 | 0.86(0.75-0.99) | 0.61(0.39-0.95) | 0.84(0.75-0.95) | 5.1 x 10^-3^ |
|  |  |  |  | II | 4379 | 1861 | 0.15/0.15 | 1.01(0.89-1.15) | 1.03(0.71-1.50) | 1.01(0.91-1.13) | 0.8 |
|  |  |  |  | Combined | 6417 | 3880 | 0.15/0.16 | 0.90(0.82-0.99) | 0.84(0.64-1.10) | 0.91(0.84-0.98) | 0.01 |
| rs11957493 | 5 | 21452916 | G/A | I | 1926 | 1934 | 0.37/0.34 | 1.27(1.11-1.45) | 1.20(0.97-1.48) | 1.15(1.04-1.26) | 4.6 x 10^-3^ |
|  |  |  |  | II | 4329 | 1857 | 0.35/0.35 | 1.02(0.91-1.15) | 1.01(0.85-1.20) | 1.01(0.93-1.10) | 0.79 |
|  |  |  |  | Combined | 6255 | 3791 | 0.36/0.34 | 1.10(1.01-1.20) | 1.10(0.96-1.25) | 1.06(1.00-1.13) | 0.05 |
| rs16867551 | 5 | 88670718 | C/A | I | 1918 | 1931 | 0.22/0.20 | 1.18(1.03-1.35) | 1.41(1.01-1.95) | 1.18(1.06-1.32) | 2.9 x 10^-3^ |
|  |  |  |  | II | 4346 | 1854 | 0.22/0.20 | 1.11(0.98-1.24) | 1.05(0.80-1.37) | 1.07(0.97-1.18) | 0.15 |
|  |  |  |  | Combined | 6264 | 3785 | 0.22/0.20 | 1.13(1.03-1.23) | 1.19(0.97-1.47) | 1.11(1.04-1.19) | 3.2 x 10^-3^ |
| rs1420864 | 5 | 103445285 | C/T | I | 2030 | 2050 | 0.39/0.36 | 1.19(1.04-1.36) | 1.28(1.05-1.55) | 1.14(1.04-1.25) | 3.6 x 10^-3^ |
|  |  |  |  | II | 4154 | 1753 | 0.38/0.38 | 0.94(0.83-1.06) | 1.03(0.87-1.22) | 1.00(0.92-1.08) | 0.92 |
|  |  |  |  | Combined | 6184 | 3803 | 0.38/0.37 | 1.02(0.93-1.11) | 1.14(1.00-1.29) | 1.05(0.99-1.12) | 0.08 |
| rs13206199 | 6 | 167683654 | C/T | I | 1926 | 1930 | 0.42/0.38 | 1.14(0.99-1.31) | 1.37(1.13-1.65) | 1.16(1.06-1.27) | 1.3 x 10^-3^ |
|  |  |  |  | II | 4362 | 1862 | 0.40/0.40 | 0.99(0.88-1.12) | 0.96(0.81-1.12) | 0.98(0.91-1.06) | 0.62 |
|  |  |  |  | Combined | 6288 | 3792 | 0.40/0.39 | 1.05(0.96-1.14) | 1.10(0.98-1.25) | 1.05(0.99-1.11) | 0.1 |
| rs10274271 | 7 | 94117082 | C/T | I | 1919 | 1936 | 0.25/0.28 | 0.89(0.78-1.01) | 0.67(0.52-0.87) | 0.85(0.77-0.94) | 1.7 x 10^-3^ |
|  |  |  |  | II | 4309 | 1845 | 0.27/0.27 | 0.95(0.85-1.07) | 1.12(0.89-1.39) | 1.00(0.92-1.10) | 0.92 |
|  |  |  |  | Combined | 6228 | 3781 | 0.26/0.28 | 0.93(0.86-1.01) | 0.91(0.78-1.07) | 0.94(0.89-1.01) | 0.08 |
| rs1806667 | 7 | 133376632 | G/T | I | 2046 | 2056 | 0.41/0.38 | 1.14(1.00-1.30) | 1.34(1.12-1.61) | 1.15(1.06-1.26) | 1.4 x 10^-3^ |
|  |  |  |  | II | 4308 | 1848 | 0.41/0.40 | 0.95(0.84-1.07) | 1.07(0.90-1.26) | 1.01(0.94-1.10) | 0.71 |
|  |  |  |  | Combined | 6354 | 3904 | 0.41/0.39 | 1.03(0.95-1.13) | 1.19(1.05-1.34) | 1.08(1.02-1.14) | 0.01 |
| rs2740873 | 8 | 3909229 | A/C | I | 2070 | 2084 | 0.15/0.18 | 0.84(0.73-0.96) | 0.70(0.48-1.03) | 0.84(0.75-0.94) | 3.1 x 10^-3^ |
|  |  |  |  | II | 4278 | 1787 | 0.17/0.16 | 0.99(0.87-1.13) | 1.38(1.02-1.85) | 1.07(0.96-1.18) | 0.21 |
|  |  |  |  | Combined | 6348 | 3871 | 0.17/0.17 | 0.90(0.82-0.98) | 1.17(0.94-1.47) | 0.97(0.90-1.04) | 0.38 |
| rs6982066 | 8 | 16012684 | C/T | I | 2048 | 2054 | 0.14/0.17 | 0.84(0.73-0.96) | 0.51(0.32-0.82) | 0.80(0.71-0.91) | 5.6 x 10^-4^ |
|  |  |  |  | II | 4382 | 1859 | 0.16/0.16 | 0.96(0.85-1.09) | 1.08(0.76-1.53) | 0.98(0.89-1.09) | 0.77 |
|  |  |  |  | Combined | 6430 | 3913 | 0.15/0.16 | 0.91(0.83-0.99) | 0.88(0.68-1.15) | 0.92(0.85-0.99) | 0.03 |
| rs17731149 | 8 | 134857676 | G/C | I | 2058 | 2068 | 0.24/0.21 | 1.25(1.10-1.42) | 1.26(0.95-1.68) | 1.19(1.07-1.32) | 9.2 x 10^-4^ |
|  |  |  |  | II | 4333 | 1861 | 0.22/0.22 | 0.96(0.85-1.08) | 0.94(0.73-1.21) | 0.96(0.88-1.06) | 0.43 |
|  |  |  |  | Combined | 6391 | 3929 | 0.23/0.22 | 1.06(0.97-1.15) | 1.05(0.87-1.27) | 1.05(0.98-1.12) | 0.2 |
| rs6560285 | 9 | 74339851 | T/C | I | 2073 | 2084 | 0.43/0.47 | 0.89(0.78-1.03) | 0.77(0.64-0.91) | 0.88(0.81-0.96) | 3.2 x 10^-3^ |
|  |  |  |  | II | 4362 | 1863 | 0.44/0.45 | 0.96(0.84-1.08) | 0.93(0.79-1.08) | 0.96(0.89-1.04) | 0.33 |
|  |  |  |  | Combined | 6435 | 3947 | 0.44/0.46 | 0.92(0.84-1.01) | 0.84(0.75-0.95) | 0.92(0.87-0.97) | 3.4 x 10^-3^ |
| rs10761309 | 9 | 95793543 | T/C | I | 1926 | 1932 | 0.35/0.38 | 0.89(0.78-1.02) | 0.75(0.61-0.92) | 0.87(0.80-0.96) | 4.3 x 10^-3^ |
|  |  |  |  | II | 4310 | 1852 | 0.36/0.36 | 1.06(0.94-1.19) | 0.97(0.82-1.15) | 1.00(0.93-1.09) | 0.91 |
|  |  |  |  | Combined | 6236 | 3784 | 0.36/0.37 | 0.97(0.89-1.06) | 0.87(0.77-0.99) | 0.94(0.89-1.00) | 0.05 |
| rs10159718 | 10 | 1129183 | G/A | I | 2072 | 2084 | 0.45/0.48 | 0.84(0.72-0.96) | 0.75(0.63-0.89) | 0.86(0.79-0.94) | 9.5 x 10^-4^ |
|  |  |  |  | II | 4328 | 1851 | 0.47/0.47 | 0.97(0.86-1.11) | 1.01(0.86-1.18) | 1.00(0.93-1.08) | 0.99 |
|  |  |  |  | Combined | 6400 | 3935 | 0.46/0.48 | 0.92(0.84-1.01) | 0.89(0.79-0.99) | 0.94(0.89-1.00) | 0.03 |
| rs1218370 | 10 | 14367180 | T/G | I | 1927 | 1938 | 0.42/0.45 | 0.87(0.75-1.00) | 0.75(0.62-0.90) | 0.86(0.79-0.95) | 1.6 x 10^-3^ |
|  |  |  |  | II | 4324 | 1847 | 0.44/0.44 | 1.00(0.88-1.13) | 1.03(0.88-1.21) | 1.01(0.94-1.09) | 0.76 |
|  |  |  |  | Combined | 6251 | 3785 | 0.43/0.45 | 0.95(0.87-1.04) | 0.91(0.81-1.02) | 0.95(0.90-1.01) | 0.09 |
| rs10827927 | 10 | 20348662 | T/G | I | 2070 | 2082 | 0.36/0.40 | 0.85(0.74-0.97) | 0.68(0.56-0.82) | 0.83(0.76-0.91) | 4.8 x 10^-5^ |
|  |  |  |  | II | 4365 | 1858 | 0.39/0.39 | 1.00(0.88-1.12) | 1.02(0.86-1.20) | 1.01(0.93-1.09) | 0.89 |
|  |  |  |  | Combined | 6435 | 3940 | 0.38/0.40 | 0.93(0.85-1.02) | 0.87(0.77-0.98) | 0.93(0.88-0.99) | 0.02 |
| rs5026308 | 10 | 130509357 | A/G | I | 2071 | 2083 | 0.06/0.09 | 0.64(0.54-0.77) | 1.03(0.45-2.33) | 0.69(0.58-0.81) | 7.8 x 10^-6^ |
|  |  |  |  | II | 4380 | 1865 | 0.08/0.08 | 0.96(0.83-1.12) | 1.01(0.54-1.89) | 0.97(0.84-1.12) | 0.68 |
|  |  |  |  | Combined | 6451 | 3948 | 0.07/0.09 | 0.82(0.73-0.92) | 1.08(0.66-1.77) | 0.85(0.77-0.95) | 2.4 x 10^-3^ |
| rs10767369 | 11 | 25356940 | G/A | I | 2056 | 2061 | 0.12/0.16 | 0.74(0.64-0.85) | 0.48(0.28-0.82) | 0.73(0.64-0.83) | 1.7 x 10^-6^ |
|  |  |  |  | II | 4337 | 1857 | 0.13/0.13 | 0.94(0.82-1.07) | 1.11(0.72-1.71) | 0.97(0.86-1.09) | 0.58 |
|  |  |  |  | Combined | 6393 | 3918 | 0.12/0.14 | 0.83(0.75-0.91) | 0.83(0.60-1.13) | 0.85(0.78-0.92) | 7.6 x 10^-5^ |
| rs5017351 | 11 | 25410058 | T/C | I | 1910 | 1911 | 0.08/0.11 | 0.78(0.66-0.92) | 0.35(0.15-0.84) | 0.75(0.64-0.88) | 3.0 x 10^-4^ |
|  |  |  |  | II | 4378 | 1860 | 0.09/0.09 | 0.94(0.82-1.09) | 1.32(0.67-2.61) | 0.97(0.85-1.11) | 0.69 |
|  |  |  |  | Combined | 6288 | 3771 | 0.09/0.10 | 0.86(0.77-0.96) | 0.81(0.50-1.29) | 0.87(0.78-0.96) | 4.3 x 10^-3^ |
| rs4127353 | 11 | 57882196 | T/C | I | 2050 | 2060 | 0.15/0.18 | 0.78(0.68-0.90) | 0.62(0.42-0.91) | 0.79(0.70-0.88) | 5.7 x 10^-5^ |
|  |  |  |  | II | 4356 | 1862 | 0.17/0.18 | 0.98(0.87-1.10) | 0.82(0.59-1.14) | 0.95(0.86-1.06) | 0.36 |
|  |  |  |  | Combined | 6406 | 3922 | 0.16/0.18 | 0.91(0.83-0.99) | 0.74(0.58-0.94) | 0.89(0.83-0.96) | 2.8 x 10^-3^ |
| rs11229410 | 11 | 57926867 | C/T | I | 2070 | 2082 | 0.16/0.18 | 0.83(0.72-0.95) | 0.61(0.42-0.88) | 0.81(0.72-0.91) | 4.1 x 10^-4^ |
|  |  |  |  | II | 4369 | 1861 | 0.17/0.18 | 0.99(0.88-1.11) | 0.87(0.64-1.20) | 0.97(0.88-1.07) | 0.55 |
|  |  |  |  | Combined | 6439 | 3943 | 0.17/0.18 | 0.93(0.85-1.02) | 0.75(0.60-0.95) | 0.91(0.84-0.98) | 0.01 |
| rs4453257 | 11 | 79491924 | C/T | I | 2072 | 2084 | 0.44/0.47 | 0.84(0.73-0.97) | 0.76(0.64-0.91) | 0.87(0.80-0.95) | 1.5 x 10^-3^ |
|  |  |  |  | II | 4326 | 1860 | 0.46/0.46 | 1.01(0.89-1.14) | 1.05(0.90-1.23) | 1.03(0.95-1.11) | 0.53 |
|  |  |  |  | Combined | 6398 | 3944 | 0.45/0.46 | 0.95(0.86-1.04) | 0.92(0.82-1.03) | 0.96(0.90-1.01) | 0.12 |
| rs11618012 | 13 | 34237417 | C/A | I | 2070 | 2075 | 0.20/0.23 | 0.93(0.82-1.06) | 0.61(0.45-0.84) | 0.87(0.78-0.97) | 0.01 |
|  |  |  |  | II | 4327 | 1846 | 0.21/0.21 | 0.99(0.88-1.11) | 1.10(0.84-1.43) | 1.01(0.92-1.11) | 0.81 |
|  |  |  |  | Combined | 6397 | 3921 | 0.21/0.22 | 0.95(0.87-1.03) | 0.87(0.72-1.06) | 0.94(0.88-1.01) | 0.09 |
| rs7333229 | 13 | 59109499 | T/C | I | 1901 | 1902 | 0.19/0.22 | 0.78(0.68-0.89) | 0.74(0.53-1.04) | 0.81(0.72-0.91) | 2.4 x 10^-4^ |
|  |  |  |  | II | 4372 | 1864 | 0.19/0.20 | 0.92(0.82-1.04) | 0.90(0.68-1.20) | 0.93(0.85-1.03) | 0.17 |
|  |  |  |  | Combined | 6273 | 3766 | 0.19/0.21 | 0.84(0.77-0.92) | 0.82(0.67-1.02) | 0.87(0.81-0.93) | 9.8 x 10^-5^ |
| rs1112044 | 13 | 99682492 | G/T | I | 1928 | 1938 | 0.25/0.29 | 0.79(0.69-0.90) | 0.72(0.56-0.94) | 0.82(0.74-0.91) | 1.6 x 10^-4^ |
|  |  |  |  | II | 4362 | 1857 | 0.27/0.27 | 1.03(0.92-1.15) | 0.96(0.78-1.19) | 1.00(0.92-1.09) | 0.96 |
|  |  |  |  | Combined | 6290 | 3795 | 0.26/0.28 | 0.91(0.84-0.99) | 0.88(0.75-1.04) | 0.93(0.87-0.99) | 0.02 |
| rs2476222 | 13 | 101333809 | T/C | I | 2062 | 2071 | 0.30/0.33 | 0.90(0.79-1.02) | 0.76(0.61-0.94) | 0.88(0.80-0.97) | 7.1 x 10^-3^ |
|  |  |  |  | II | 4315 | 1838 | 0.31/0.31 | 0.98(0.88-1.10) | 0.98(0.80-1.18) | 0.99(0.91-1.07) | 0.74 |
|  |  |  |  | Combined | 6377 | 3909 | 0.31/0.32 | 0.94(0.86-1.02) | 0.86(0.75-0.99) | 0.93(0.88-0.99) | 0.02 |
| rs1123204 | 14 | 36289020 | G/A | I | 1926 | 1937 | 0.28/0.31 | 0.92(0.81-1.05) | 0.72(0.57-0.91) | 0.88(0.80-0.97) | 9.8x 10^-3^ |
|  |  |  |  | II | 4334 | 1861 | 0.30/0.30 | 0.98(0.87-1.10) | 0.98(0.81-1.20) | 0.99(0.91-1.07) | 0.77 |
|  |  |  |  | Combined | 6260 | 3798 | 0.29/0.30 | 0.96(0.88-1.04) | 0.90(0.77-1.04) | 0.95(0.89-1.01) | 0.12 |
| rs4383110 | 15 | 31823762 | T/C | I | 2071 | 2083 | 0.17/0.19 | 0.86(0.75-0.98) | 0.67(0.47-0.94) | 0.84(0.75-0.94) | 2.5 x 10^-3^ |
|  |  |  |  | II | 4322 | 1847 | 0.19/0.18 | 1.02(0.91-1.15) | 1.12(0.83-1.52) | 1.04(0.94-1.14) | 0.48 |
|  |  |  |  | Combined | 6393 | 3930 | 0.18/0.19 | 0.96(0.87-1.04) | 0.90(0.73-1.12) | 0.95(0.89-1.02) | 0.19 |
| rs4451969 | 16 | 11291020 | C/T | I | 2071 | 2084 | 0.25/0.22 | 1.19(1.04-1.35) | 1.36(1.03-1.79) | 1.18(1.06-1.30) | 1.8 x 10^-3^ |
|  |  |  |  | II | 4321 | 1854 | 0.24/0.24 | 0.98(0.88-1.10) | 0.85(0.67-1.07) | 0.95(0.87-1.04) | 0.28 |
|  |  |  |  | Combined | 6392 | 3938 | 0.24/0.23 | 1.07(0.99-1.17) | 1.05(0.88-1.25) | 1.05(0.98-1.12) | 0.16 |
| rs17821987 | 16 | 58959843 | T/C | I | 1928 | 1938 | 0.10/0.08 | 1.23(1.03-1.46) | 1.61(0.80-3.25) | 1.24(1.06-1.45) | 8.1 x 10^-3^ |
|  |  |  |  | II | 4341 | 1863 | 0.09/0.08 | 1.22(1.04-1.42) | 0.62(0.33-1.15) | 1.13(0.98-1.30) | 0.1 |
|  |  |  |  | Combined | 6269 | 3801 | 0.09/0.08 | 1.19(1.07-1.34) | 0.90(0.57-1.44) | 1.15(1.03-1.27) | 9.6 x 10^-3^ |
| rs16975392 | 18 | 10802573 | A/G | I | 1926 | 1937 | 0.41/0.45 | 0.83(0.72-0.96) | 0.72(0.60-0.87) | 0.85(0.77-0.93) | 2.8 x 10^-4^ |
|  |  |  |  | II | 4364 | 1861 | 0.44/0.44 | 0.95(0.84-1.07) | 0.95(0.81-1.11) | 0.97(0.90-1.05) | 0.43 |
|  |  |  |  | Combined | 6290 | 3798 | 0.43/0.45 | 0.91(0.83-1.00) | 0.86(0.77-0.97) | 0.93(0.87-0.98) | 9.1 x 10^-3^ |
| rs4147513 | 18 | 44200273 | T/C | I | 1927 | 1937 | 0.32/0.36 | 0.82(0.72-0.94) | 0.72(0.59-0.89) | 0.84(0.76-0.92) | 2.9 x 10^-4^ |
|  |  |  |  | II | 4362 | 1863 | 0.34/0.32 | 1.11(0.99-1.25) | 1.37(1.13-1.67) | 1.15(1.06-1.25) | 1.3 x 10^-3^ |
|  |  |  |  | Combined | 6289 | 3800 | 0.34/0.34 | 0.98(0.90-1.07) | 1.00(0.87-1.14) | 0.99(0.93-1.06) | 0.82 |
| rs6100755 | 20 | 58159233 | G/A | I | 1926 | 1936 | 0.46/0.50 | 0.88(0.76-1.03) | 0.72(0.60-0.86) | 0.85(0.78-0.93) | 4.2 x 10^-4^ |
|  |  |  |  | II | 4318 | 1854 | 0.48/0.47 | 1.03(0.90-1.17) | 1.06(0.91-1.24) | 1.03(0.95-1.11) | 0.45 |
|  |  |  |  | Combined | 6244 | 3790 | 0.47/0.49 | 0.96(0.87-1.05) | 0.90(0.80-1.01) | 0.95(0.90-1.00) | 0.07 |
| rs2828070 | 21 | 23622428 | G/A | I | 2072 | 2082 | 0.35/0.30 | 1.18(1.04-1.35) | 1.67(1.35-2.08) | 1.25(1.14-1.37) | 2.5 x 10^-6^ |
|  |  |  |  | II | 4337 | 1848 | 0.32/0.32 | 0.96(0.85-1.07) | 1.03(0.85-1.24) | 0.99(0.92-1.08) | 0.88 |
|  |  |  |  | Combined | 6409 | 3930 | 0.33/0.31 | 1.03(0.95-1.13) | 1.26(1.10-1.45) | 1.09(1.03-1.16) | 5.0 x 10^-3^ |
| rs200674 | 21 | 23628441 | G/A | I | 2059 | 2070 | 0.34/0.30 | 1.16(1.02-1.32) | 1.61(1.30-2.01) | 1.23(1.12-1.35) | 2.2 x 10^-5^ |
|  |  |  |  | II | 4347 | 1857 | 0.32/0.32 | 0.95(0.84-1.06) | 1.03(0.86-1.25) | 0.99(0.91-1.08) | 0.83 |
|  |  |  |  | Combined | 6406 | 3927 | 0.32/0.31 | 1.02(0.93-1.10) | 1.24(1.08-1.42) | 1.08(1.01-1.14) | 0.02 |
| rs5756968 | 22 | 36925186 | C/T | I | 2073 | 2084 | 0.25/0.27 | 0.86(0.76-0.98) | 0.75(0.58-0.96) | 0.86(0.78-0.95) | 3.3 x 10^-3^ |
|  |  |  |  | II | 4328 | 1854 | 0.24/0.25 | 0.94(0.84-1.06) | 1.08(0.86-1.36) | 0.99(0.91-1.08) | 0.82 |
|  |  |  |  | Combined | 6401 | 3938 | 0.24/0.26 | 0.89(0.82-0.96) | 0.91(0.77-1.07) | 0.92(0.86-0.98) | 0.01 |

a SNPs selected for Stage III evaluation are not presented in this table.

b effect allele/reference allele.

c frequency of effect allele in cases/controls.

d Adjusted for age and principal components for ancestry in Stage I, and age in Stage II and combined analyses.
