# Supplementary material for: Identification of a Functional Genetic Variant at 16q12.1 for Breast Cancer Risk: Results from the Asia Breast Cancer Consortium
Source: PLoS Genet. 2010 Jun 24;6(6):e1001002. doi: 10.1371/journal.pgen.1001002 (PMC2891809; doi:10.1371/journal.pgen.1001002)
Supplement: Table S3 — Results for four SNPs selected for Stage III evaluation by study. (0.09 MB DOC) [file pgen.1001002.s003.doc]

Table S3. Results for four SNPs selected for Stage III evaluation by study.

|  |  |  |  |  |  |  | OR(95%CI)c | |  |
| --- | --- | --- | --- | --- | --- | --- | --- | --- | --- |
| SNP (chr) | position | allele^a^ | Study | No of cases | No of controls | Frequency^b^ | Heterozygous | Homozygous | P for trend |
| rs10479046 (5q31.1) | 132945455 | C/T | Shanghai | 6,391 | 3,937 | 0.719/0.698 | 1.09(0.94-1.27) | 1.22(1.06-1.42) | 7.5 x10^-4^ |
|  |  |  | Tianjin | 1,523 | 1,582 | 0.701/0.693 | 0.92(0.71-1.19) | 1.01(0.78-1.30) | 0.48 |
|  |  |  | Nanjing | NA | NA | NA | NA | NA | NA |
|  |  |  | Taiwan | 1,024 | 1,037 | 0.709/0.701 | 1.03(0.73-1.45) | 1.04(0.74-1.46) | 0.82 |
|  |  |  | Hong Kong | 411 | 599 | 0.698/0.687 | 1.40(0.88-2.24) | 1.32(0.83-2.11) | 0.53 |
|  |  |  | Nagoya, Japan | NA | NA | NA | NA | NA | NA |
|  |  |  | Nagano, Japan | 403 | 403 | 0.699/0.676 | 0.87(0.53-1.42) | 1.07(0.66-1.76) | 0.34 |
|  |  |  | MEC | 532 | 512 | 0.717/0.709 | 1.15(0.70-1.91) | 1.11(0.68-1.83) | 0.91 |
|  |  |  | All Asians | 10,284 | 8,070 | 0.714/0.696 | 1.06(0.95-1.19) | 1.16(1.04-1.29) | 7.3 x10^-4^ |
| rs3829849 (9q33.3) | 128430621 | T/C | Shanghai | 6,398 | 3,952 | 0.106/0.089 | 1.24(1.11-1.38) | 1.34(0.89-2.01) | 4.4 x10^-5^ |
|  |  |  | Tianjin | 1,524 | 1,582 | 0.113/0.111 | 1.11(0.93-1.33) | 0.62(0.33-1.16) | 0.76 |
|  |  |  | Nanjing | 1,438 | 1,438 | 0.106/0.097 | 1.07(0.89-1.29) | 1.48(0.69-3.21) | 0.29 |
|  |  |  | Taiwan | 1,041 | 1,032 | 0.09/0.086 | 0.94(0.74-1.20) | 7.09(1.58-31.74) | 0.47 |
|  |  |  | Hong Kong | 416 | 612 | 0.107/0.118 | 0.94(0.69-1.29) | 0.52(0.16-1.64) | 0.39 |
|  |  |  | Nagoya, Japan | NA | NA | NA | NA | NA | NA |
|  |  |  | Nagano, Japan | 403 | 403 | 0.143/0.169 | 0.95(0.69-1.31) | 0.36(0.14-0.94) | 0.16 |
|  |  |  | MEC | 533 | 508 | 0.187/0.165 | 1.28(0.96-1.70) | 1.04(0.53-2.03) | 0.19 |
|  |  |  | All Asians | 11,753 | 9,527 | 0.111/0.103 | 1.14(1.06-1.22) | 1.06(0.83-1.36) | 1.1 x10^-3^ |
| rs7966820 (12q24.1) | 108786653 | T/C | Shanghai | 6,414 | 3,930 | 0.148/0.13 | 1.12(1.02-1.23) | 1.60(1.19-2.16) | 4.0 x10^-4^ |
|  |  |  | Tianjin | NA | NA | NA | NA | NA | NA |
|  |  |  | Nanjing | NA | NA | NA | NA | NA | NA |
|  |  |  | Taiwan | 1,049 | 1,039 | 0.14/0.148 | 0.83(0.68-1.02) | 1.22(0.62-2.38) | 0.22 |
|  |  |  | Hong Kong | 417 | 614 | 0.144/0.156 | 0.84(0.63-1.13) | 1.07(0.51-2.23) | 0.44 |
|  |  |  | Nagoya, Japan | NA | NA | NA | NA | NA | NA |
|  |  |  | Nagano, Japan | 403 | 403 | 0.19/0.213 | 0.93(0.69-1.26) | 0.56(0.26-1.19) | 0.23 |
|  |  |  | MEC | 533 | 510 | 0.257/0.231 | 1.18(0.90-1.54) | 1.03(0.60-1.77) | 0.4 |
|  |  |  | All Asians | 8,816 | 6,496 | 0.155/0.148 | 1.05(0.98-1.14) | 1.29(1.04-1.59) | 0.02 |
| rs4784227 (16q12.1) | 51156689 | T/C | Shanghai | 6,346 | 3,921 | 0.284/0.244 | 1.24(1.14-1.35) | 1.50(1.28-1.77) | 2.1 x10^-10^ |
|  |  |  | Tianjin | 1,520 | 1,583 | 0.299/0.25 | 1.38(1.19-1.61) | 1.44(1.09-1.90) | 1.6 x10^-5^ |
|  |  |  | Nanjing | 1,437 | 1,437 | 0.289/0.25 | 1.15(0.99-1.34) | 1.67(1.24-2.25) | 7.8 x10^-4^ |
|  |  |  | Taiwan | 1,003 | 1,010 | 0.294/0.238 | 1.37(1.14-1.65) | 1.69(1.19-2.42) | 6.0 x10^-4^ |
|  |  |  | Hong Kong | 456 | 644 | 0.288/0.259 | 1.17(0.91-1.51) | 1.31(0.84-2.05) | 0.13 |
|  |  |  | Nagoya, Japan | 640 | 631 | 0.293/0.226 | 1.61(1.28-2.03) | 1.61(1.01-2.56) | 1.1 x10^-4^ |
|  |  |  | Nagano, Japan | 403 | 403 | 0.303/0.217 | 1.48(1.11-1.98) | 2.99(1.59-5.62) | 8.3 x10^-5^ |
|  |  |  | MEC | 531 | 511 | 0.263/0.248 | 1.21(0.94-1.56) | 0.94(0.56-1.57) | 0.42 |
|  |  |  | All Asians | 12,336 | 10,140 | 0.288/0.244 | 1.28(1.21-1.35) | 1.52(1.37-1.69) | 3.2 x10^-25^ |

a Effect allele/reference allele.

b Effect allele frequency in cases/controls.

c Within each study, adjusted for age, and in the combined analyses, age, and study site were adjusted.
