# Supplementary material for: Identification of a Functional Genetic Variant at 16q12.1 for Breast Cancer Risk: Results from the Asia Breast Cancer Consortium
Source: PLoS Genet. 2010 Jun 24;6(6):e1001002. doi: 10.1371/journal.pgen.1001002 (PMC2891809; doi:10.1371/journal.pgen.1001002)
Supplement: Table S4 — Haplotype analyses of the four SNPs at 16q12 with breast cancer risk in Stages I and II. (0.05 MB DOC) [file pgen.1001002.s004.doc]

Table S4. Haplotype analyses of the four SNPs at 16q12 with breast cancer risk in Stages I and II.

| Haplotype | rs8051542 | rs12443621 | rs3803662 | rs4784227 | Frequency^a^ | OR | 95% CI | |
| --- | --- | --- | --- | --- | --- | --- | --- | --- |
| 1 | C | G | A | C | 0.20/0.22 | 1.00 | (reference) | |
| 2 | C | A | A | C | 0.18/0.17 | 1.16 | 0.97 | 1.37 |
| 3 | C | A | G | C | 0.18/0.19 | 1.01 | 0.85 | 1.2 |
| 4 | C | G | G | C | 0.12/0.14 | 0.96 | 0.8 | 1.16 |
| 5 | T | G | A | T | 0.16/0.14 | 1.24 | 1.03 | 1.48 |
| 6 | C | G | A | T | 0.06/0.04 | 1.49 | 1.13 | 1.95 |
| 7 | C | A | A | T | 0.05/0.05 | 1.13 | 0.87 | 1.48 |
| 8 | C | G | G | T | 0.01/0.01 | 1.34 | 0.77 | 2.32 |
| 9 | T | G | A | C | 0.01/0.02 | 0.86 | 0.55 | 1.34 |

a Frequency in cases/controls.
