# Supplementary material for: Identification of a Functional Genetic Variant at 16q12.1 for Breast Cancer Risk: Results from the Asia Breast Cancer Consortium
Source: PLoS Genet. 2010 Jun 24;6(6):e1001002. doi: 10.1371/journal.pgen.1001002 (PMC2891809; doi:10.1371/journal.pgen.1001002)
Supplement: Text S1 — Study participants. (0.11 MB DOC) [file pgen.1001002.s005.doc]

**Supplemental Text S1 Study Participants**

**Shanghai Breast Cancer Study (SBCS):** The SBCS is a population-based, case-control study conducted in urban Shanghai, the largest commercial center in China [1, 2]. For the SBCS-I, subjects were recruited between 1996 and 1998. Through a rapid case-ascertainment system and the population-based Shanghai Cancer Registry, 1,602 eligible breast cancer cases diagnosed during the study period were identified, of which 1,459 cases (91.1%) completed in-person interviews. Cancer diagnoses for all patients were reviewed and confirmed by two senior pathologists. Controls were randomly selected from the general population using the Shanghai Resident Registry, a population registry containing demographic information for all residents of urban Shanghai. The inclusion criteria for controls were identical to those for cases with the exception of a breast cancer diagnosis. Of the 1,724 eligible controls, 1,556 (90.3%) completed in-person interviews. A structured questionnaire was used to elicit detailed information on demographic factors and known/suspected risk factors for breast cancer. All participants were measured for their current weight, height, and circumference of the waist and hips. All interviews were tape-recorded and reviewed by the field supervisor and quality control staff to monitor the quality of interview data. Blood samples (10 ml from each woman) were obtained from 1,193 (82%) cases and 1,310 (84%) controls who completed the in-person interview. A sample of exfoliated buccal cells was obtained using cotton swabs from virtually all study participants who did not provide a blood sample. Because DNA yield from buccal cell samples collected in cotton swabs is low, the current study is limited to those who provided a blood sample.

#### Using a protocol similar to the SBCS-I, the SBCS-II recruited 1,989 incident breast cancer cases and 1,989 community controls between 2002 and 2005 with a response rate of 83.7% and 70.4%, respectively. Similar to subject recruitment in the SBCS-I, the majority of newly-recruited cases (n=1,932, 97.1%) and controls (n=1,857, 93.4%) provided a blood sample or an exfoliated buccal cell sample to the study. The mouthwash method used in the study was modified from that reported initially by Lum and Le Marchand [3] and provided, on average, approximately 34 μg of DNA per sample. With the exception of age, eligibility criteria for study participation were identical for SBCS-I and SBCS-II. The age range was expanded from 25 to 65 years in SBCS-I to 25 to 70 years in SBCS-II.

**Shanghai Breast Cancer Survival Study (SBCSS)** **and** **Shanghai Endometrial Cancer Study (SECS):** The SBCSS also used the population-based Shanghai Cancer Registry to identify newly-diagnosed breast cancer cases for the study [2]. A total of 6,303 cases were diagnosed between April 1, 2002 and December 31, 2006 and approached for the study approximately six months after cancer diagnosis; 5,046 were recruited (response rate: 80.1%). In-person interviews were conducted to collect information on known breast cancer risk factors and anthropometrics by using a protocol and questionnaire similar to that used in the SBCS. Buccal cell samples were collected from 96% of study participants using the modified mouthwash method described above. Because of a time overlap in subject recruitment in the SBCS-II and the SBCSS, 1,469 breast cancer patients participated in both studies. The remaining 3,466 SBCSS cases were included in the current study.

Controls for this group of cases were derived from the SECS [2], a population-based case-control study conducted between 1997 and 2003, a time period that overlapped with the SBCS and SBCSS. With the exception of a few questions related specifically to breast or endometrial cancer risk, the questionnaires used in the SECS and the SBCS were virtually identical. Using a protocol similar to the one used in the SBCS, eligible cases were identified through the population-based Shanghai Cancer Registry and controls were randomly selected from the general population of Shanghai using the Shanghai Resident Registry and were age frequency matched to cases. Women with a history of cancer or hysterectomy were not eligible. In-person interviews were conducted by trained interviewers to collect detailed information on demographic factors as well as known and suspected risk factors. Of the study participants who completed an in-person interview, 1,039 controls provided a blood sample or buccal cell sample using the mouthwash method, and these women were included in the current study

**Tianjin Study** [4]: This hospital-based case-control study included 1,532 breast cancer patients and 1,583 healthy female controls. Patients were histopathologically diagnosed with primary breast cancer and were consecutively recruited from the Department of Breast Surgery of Tianjin Cancer Institute and Hospital, China, between December 2004 and December 2008. Age (±5 years) frequency-matched controls were recruited from women who attended health screenings at the Center of Health Examination in the same hospital during the same period and had no evidence of cancer. Controls with diseases of the cardiovascular, respiratory, digestive, urinary, reproductive, or endocrine systems were also excluded. All breast cancer patients and controls were genetically unrelated, ethnic Han Chinese women who were permanent residents of the urban area of Tianjin. ER and PR status was determined using immunohistochemical analysis by breast cancer pathologists in the Department of Pathology. After giving informed consent, each participant was interviewed face-to-face by trained interviewers using a pre-tested questionnaire to obtain information on demographic data, menstrual and reproductive history, lifestyle, environmental exposures, and family history of cancer. After the interview, a 5-ml venous blood sample was collected from each participant and used for DNA extraction and genotyping. The study protocol was approved by the Tianjin Cancer Institute and Hospital review committee.

**Nanjing Study** [5, 6]: This hospital-based case-control study included 1,446 breast cancer cases and 1,439 cancer-free controls. Incident breast cancer patients were consecutively recruited from the First Affiliated Hospital of Nanjing Medical University, the Cancer Hospital of Jiangsu Province and the Gulou Hospital, Nanjing, China, between January 2004 and July 2008. Exclusion criteria included self-reported prior history of cancer, metastasized cancer from other organs, and previous radiotherapy or chemotherapy. All included breast cancer cases were newly diagnosed and histopathologically confirmed without restrictions of age. Cancer-free controls, frequency-matched to the cases on age (±5 years) and residential area (urban or rural), were randomly selected from a cohort of >30,000 participants in a community-based screening program for non-infectious diseases conducted from 2004 to 2006 in Jiangsu Province, China. All participants were genetically unrelated, ethnic Han Chinese women. After providing informed consent, each woman was interviewed face-to-face by trained interviewers using a standard questionnaire to obtain information on demographic characteristics, menstrual and reproductive history, environmental exposure history, and family history of any cancer in first-degree relatives (parents, siblings, and children). Data collection methods were similar for cases and controls. After the interview, each subject provided 5 ml of venous blood. The ER and PR status of breast cancers was determined from the results of immunohistochemical examinations recorded in the medical records of the hospitals. This study was approved by the institutional review board of Nanjing Medical University.

**Taiwan Study** [7, 8]: This case-control study is part of an on-going, cooperative study aimed at understanding the causes of breast cancer in Taiwan, which is characterized by low incidence, early tumor onset, hormone dependency and novel genomic alterations. The study included 1,001 female breast cancer patients and 1,013 healthy female controls. All breast cancer patients had pathologically confirmed incident primary breast cancer and were diagnosed and treated at the Tri-Service General Hospital or the Changhua Christian Hospital between March 2002 and August 2005. The participation rate was over 90%. Patients with inadequate blood specimens were excluded from the study. Women included in the study were similar to those excluded in the distribution of major breast cancer risk factors. Because these are two of the major breast cancer clinics in northern and central Taiwan, patients recruited for the study accounted for a significant proportion (~40%) of all breast cancer cases diagnosed during the study period in these regions. Controls were randomly selected from women attending the health examination clinics of the same hospitals during the same period. These women underwent a one-day comprehensive health examination (including regular breast screening using X-ray mammography and ultrasonic examination) and those showing any evidence of breast cancer, suspicious precancerous lesions of the breast, or other cancers were excluded from the control group. Almost all women (>95%) initially identified as potential controls participated in the study and the controls accounted for ~20% of all women attending the clinics; no significant differences in socioeconomic status were found between those included and those excluded from the study. Informed consent was obtained from all study participants before collection of epidemiologic data through in-person interviews. At the completion of each interview, blood was taken for DNA isolation and genotyping. Two experienced research nurses were assigned to administer a structured questionnaire to both cases and controls. The information collected included age, family history of breast cancer, age at menarche and/or menopause, history of full-term pregnancy, menopausal status, and body mass index.

**Hong Kong Study** [9]: This is a hospital-based study consisting of women with incident breast cancer, recruited during the period of June 2003 to March 2009 from patients attending follow-up surgical and oncology outpatient clinics at three major public hospitals on Hong Kong Island (Queen Mary Hospital) and Kowloon (Queen Elisabeth Hospital and Kwong Wah Hospital). All participants completed face-to-face interviews. Control participants matched for age on 10-year intervals were recruited from outpatients attending the general gynecological clinic at Queen Mary Hospital and from the Well-Women Clinic at Kwong Wah Hospital, who had no personal history of cancer. They were also questioned about any family history of breast and/or ovarian cancer. About 70% of cases and controls interviewed agreed to participate in this project. Blood samples were obtained from 517 cases and 651 controls, which were subsequently used for DNA extraction by proteinase K digestion followed by conventional phenol-chloroform-ethanol extraction. The protocol was approved by the Institutional Review Boards of the University of Hong Kong Hospital Authority, and patient consent was obtained for study participation and blood collection.

**The Multiethnic Cohort Study** **(MEC)** [10, 11]: The MEC is a population-based prospective cohort study that was initiated between 1993 and 1996 and includes subjects from various ethnic groups – African-Americans and Latinos primarily from California (mainly Los Angeles) and Native Hawaiians, Japanese-Americans, and European Americans primarily from Hawaii. State driver's license files were the primary sources used to identify study subjects in Hawaii and California. Additionally, in Hawaii, state voter's registration files were used, and in California, Health Care Financing Administration (HCFA) files were used to identify additional African American men. All participants (n = 215,251) returned a 26-page self-administered baseline questionnaire that obtained general demographic, medical, and risk factor information such as ethnicity, prior medical conditions, family history of various cancers, dietary exposures, smoking, physical activity, body mass index (BMI), and for women, reproductive history and exogenous hormone use. All participants were 45 to 75 years of age at baseline. In the cohort, incident cancer cases are identified annually through cohort linkage to population-based cancer Surveillance, Epidemiology, and End Results (SEER) registries in Hawaii and Los Angeles County as well as to the California State cancer registry. Information on stage of disease and estrogen and progesterone receptor status was also obtained through the SEER registries. Blood sample collection in the MEC began in 1994 and targeted incident breast cancer cases and a random sample of study participants to serve as controls for genetic analyses. In the present study, incident cases were defined as those diagnosed with invasive breast cancer after enrollment through December 31, 2005 in Hawaii and January 31, 2006 in California. Cases were over 45 years of age and consisted primarily of postmenopausal women. Women with a previous diagnosis of breast cancer identified by SEER or self-reported at baseline were excluded. Controls were women without a breast cancer diagnosis through December 31, 2005 in Hawaii and January 31, 2006 in California. The controls were frequency-matched to cases on ethnicity and the case's age at diagnosis in five-year intervals. The nested breast cancer case-control study consists of 541 invasive Japanese American breast cancer cases and 507 controls, and has been utilized previously for numerous candidate gene association studies in the MEC. This study was approved by the Institutional Review Boards at the University of Southern California and the University of Hawaii and informed consent was obtained from all study participants.

#### Nagano Breast Cancer Study [12]: This multicenter, hospital-based, case-control study was conducted from May 2001 to September 2005 at four hospitals in Nagano Prefecture, Japan. The cases, a consecutive series of women aged 20–74 years with newly diagnosed, histologically confirmed invasive breast cancer, were admitted to the four hospitals during the survey period. Of the 412 eligible patients, 405 (98%) agreed to participate. Healthy controls were selected from medical checkup examinees in two of the hospitals who were confirmed as not having any cancer, with one control matched for each case by age (within three years) and residential area during the study period. Among potential control subjects, one declined to participate. Written informed consent was obtained from 405 matched pairs. Because two controls refused to provide blood samples, the analysis was restricted to 403 matched pairs. Participants were asked to complete a self-administered questionnaire, which included questions on demographic characteristics, anthropometric factors, smoking habits, family history of cancer, physical activity, medical history, and menstrual and reproductive history. Dietary habits were investigated using a 136-item semi-quantitative food-frequency questionnaire (FFQ), which was developed and validated in the Japanese population. The ER and PR status of the patient’s breast cancer tissue was obtained from medical records. Hormone receptor positivity values were determined either as specified by the laboratory that performed the assay, in accordance with the laboratory’s written interpretation thereof, or both. The study protocol was approved by the institutional review board of the National Cancer Center (Tokyo, Japan).

#### Hospital-based Epidemiologic Research Program at Aichi Cancer Center (HERPACC2) [13]: This is a hospital-based, comprehensive epidemiologic research program at the Aichi Cancer Center (ACC), Japan. All first-visit outpatients 20-79 years of age at the ACC from December 2000 to November 2005 were asked to participate in the HERPACC2.  A total of 29,736 eligible patients were approached and 28,766 participated in the study, with a response rate of 96.7%. Subjects were asked to fill out a self-administered questionnaire about their lifestyle and demographic characteristics and to provide blood samples. Dietary habits were investigated using a 47-item semi-quantitative food frequency questionnaire. ER status for cases was taken from medical records. ER status is routinely determined by pathologists by using commercially based immunohistochemistry tests at the ACC. Case status was confirmed by linkage of the HERPACC2 database and the hospital-based cancer registry database. 1,850 histologically-confirmed breast cancer cases were identified and 644 were selected for the Asia Breast Cancer Consortium analysis based on availability of DNA samples. Of 14,260 non-cancer subjects in the HERPACC2 database, 644 subjects matched for age and menopausal status were randomly selected. The study protocol was approved by the institutional review board at the ACC (Nagoya, Japan).

**The Nashville Breast Health Study** **(NBHS)** [2]: The NBHS is a population-based, case-control study of breast cancer conducted in the eight-county Nashville, TN metropolitan area. Through a rapid case ascertainment system, we identified newly-diagnosed breast cancer cases through the Tennessee State Cancer Registry and five major hospitals in the city that provide medical care for breast cancer patients. Eligible cases were women diagnosed between April 1, 2001 and March 31, 2008 with invasive breast cancer or ductal carcinoma in situ, who were between the ages of 25 and 75, had no prior history of cancer other than non-melanoma skin cancer, had a resident telephone, spoke English, and who were able to provide consent to the study. Controls were identified via random digit dialing (RDD) of households in the same geographic area as cases. Eligibility criteria were the same for controls and cases, except that controls could not have a prior cancer diagnosis other than simple skin cancer. Controls were frequency matched to cases on five-year age group, race, and county of residence. Approval for this study was garnered from the Institutional Review Board of Vanderbilt University Medical Center and those of the individual collaborating institutions. All participants provided informed consent prior to enrollment in this study. Information on demographic factors, as well as known and suspected risk factors for breast cancer, was ascertained through a structured questionnaire administered via telephone interview, and through a self-administered food frequency questionnaire. All interviews were recorded for quality control and data monitoring purposes. Buccal cell samples were collected via two methods: Oragene saliva collection kits (DNA GenoteK, Ottawa, Canada) and mouthwash samples. The NBHS is an on-going study that continues to recruit eligible cases and controls. The overall response rate is estimated to be approximately 65 to 70%. Included in the current projects are 1,591 cases and 1,466 controls of European ancestry who participated in the study before August 2008.

**CGEMS (Cancer Genetic Markers of Susceptibility)**: We also used data from the National Cancer Institute CGEMS study (http://cgems.cancer.gov/data/). Individual genotyping data for 1,145 breast cancer cases and 1,142 controls nested within the prospective Nurses’ Health Study cohort were obtained through an approved data request application. These women were included in Stage I of the CGEMS breast cancer study.

References

(1) Gao YT, Shu XO, Dai Q, Potter JD, Brinton LA, et al. (2000) Association of menstrual and reproductive factors with breast cancer risk: results from the Shanghai Breast Cancer Study. Int J Cancer 87:295-300.

(2) Zheng W, Long J, Gao YT, Li C, Zheng Y, et al. (2009) Genome-wide association study identifies a new breast cancer susceptibility locus at 6q25.1. Nat Genet 41:324-328.

(3) Lum A, Le ML. (1998) A simple mouthwash method for obtaining genomic DNA in molecular epidemiological studies. Cancer Epidemiol Biomarkers Prev 7:719-724.

(4) Zhang L, Gu L, Qian B, Hao X, Zhang W, et al. (2009) Association of genetic polymorphisms of ER-alpha and the estradiol-synthesizing enzyme genes CYP17 and CYP19 with breast cancer risk in Chinese women. Breast Cancer Res Treat 114:327-338.

(5) Liang J, Chen P, Hu Z, Zhou X, Chen L, et al. (2008) Genetic variants in fibroblast growth factor receptor 2 (FGFR2) contribute to susceptibility of breast cancer in Chinese women. Carcinogenesis 29:2341-2346.

(6) Wang Y, Hu Z, Liang J, Wang Z, Tang J, et al. (2008) A tandem repeat of human telomerase reverse transcriptase (hTERT) and risk of breast cancer development and metastasis in Chinese women. Carcinogenesis 29:1197-1201.

(7) Ding SL, Yu JC, Chen ST, Hsu GC, Kuo SJ, et al. (2009) Genetic variants of BLM interact with RAD51 to increase breast cancer susceptibility. Carcinogenesis 30:43-49.

(8) Hsu HM, Wang HC, Chen ST, Hsu GC, Shen CY, et al. (2007) Breast cancer risk is associated with the genes encoding the DNA double-strand break repair Mre11/Rad50/Nbs1 complex. Cancer Epidemiol Biomarkers Prev 16:2024-2032.

(9) Chan KY, Liu W, Long JR, Yip SP, Chan SY, et al. (2009) Functional polymorphisms in the BRCA1 promoter influence transcription and are associated with decreased risk for breast cancer in Chinese women. J Med Genet 46:32-39.

(10) Haiman CA, Garcia RR, Hsu C, Xia L, Ha H, et al. (2009) Screening and association testing of common coding variation in steroid hormone receptor co-activator and co-repressor genes in relation to breast cancer risk: the Multiethnic Cohort. BMC Cancer 9:43.:43.

(11) Kolonel LN, Henderson BE, Hankin JH, Nomura AM, Wilkens LR, et al. (2000) A multiethnic cohort in Hawaii and Los Angeles: baseline characteristics. Am J Epidemiol 151:346-357.

(12) Itoh H, Iwasaki M, Hanaoka T, Kasuga Y, Yokoyama S, et al. (2009) Serum organochlorines and breast cancer risk in Japanese women: a case-control study. Cancer Causes Control 20:567-580.

(13) Hamajima N, Matsuo K, Saito T, Hirose K, Inoue M, et al. (2001) Gene-environment Interactions and Polymorphism Studies of Cancer Risk in the Hospital-based Epidemiologic Research Program at Aichi Cancer Center II (HERPACC-II). Asian Pac J Cancer Prev 2:99-107.
